# Structural networking of the developing brain: from maturation to neurosurgical implications

**DOI:** 10.3389/fnana.2023.1242757

**Published:** 2023-11-30

**Authors:** Alessandro De Benedictis, Maria Camilla Rossi-Espagnet, Luca de Palma, Silvio Sarubbo, Carlo Efisio Marras

**Affiliations:** ^1^Neurosurgery Unit, Bambino Gesù Children’s Hospital, IRCCS, Rome, Italy; ^2^Neuroradiology Unit, Bambino Gesù Children’s Hospital, IRCCS, Rome, Italy; ^3^Clinical and Experimental Neurology, Bambino Gesù Children’s Hospital, Rome, Italy; ^4^Department of Neurosurgery, Santa Chiara Hospital, Azienda Provinciale per i Servizi Sanitari (APSS), Trento, Italy

**Keywords:** brain connectome, white matter, anatomo-functional maturation, pediatric neurosurgery, structural connectivity

## Abstract

Modern neuroscience agrees that neurological processing emerges from the multimodal interaction among multiple cortical and subcortical neuronal hubs, connected at short and long distance by white matter, to form a largely integrated and dynamic network, called the brain “connectome.” The final architecture of these circuits results from a complex, continuous, and highly protracted development process of several axonal pathways that constitute the anatomical substrate of neuronal interactions. Awareness of the network organization of the central nervous system is crucial not only to understand the basis of children’s neurological development, but also it may be of special interest to improve the quality of neurosurgical treatments of many pediatric diseases. Although there are a flourishing number of neuroimaging studies of the connectome, a comprehensive vision linking this research to neurosurgical practice is still lacking in the current pediatric literature. The goal of this review is to contribute to bridging this gap. In the first part, we summarize the main current knowledge concerning brain network maturation and its involvement in different aspects of normal neurocognitive development as well as in the pathophysiology of specific diseases. The final section is devoted to identifying possible implications of this knowledge in the neurosurgical field, especially in epilepsy and tumor surgery, and to discuss promising perspectives for future investigations.

## 1 Introduction

Over the past few decades, modern neuroscientific research has advanced towards a more realistic comprehension of the anatomo-functional basis of brain processing (McIntosh, 2000; Bressler and Tognoli, 2006; Reijneveld et al., 2007; De Benedictis and Duffau, 2011; Sarubbo et al., 2015, 2020). As opposed to the classical localizationist model of a rigid anatomo-functional dependence between structure and function, growing evidence supports a more dynamic organization of the Central Nervous System (CNS), as a complex network consisting of highly distributed cortical neuronal hubs connected at short and long distance by widely integrated axonal sub-circuits, forming the so-called brain “connectome” (Collin and van den Heuvel, 2013; Sporns, 2013; Castellanos et al., 2014; Sporns, 2015). According to this framework, neurological function emerges from the multimodal, parallel, and often redundant interaction among multiple essential functional epicenters, regulated and modulated by other brain regions (De Benedictis and Duffau, 2011; Duffau, 2018; Herbet and Duffau, 2020; Duffau, 2021a,b).

The development of a human being undergoes a constant and irreversible process of biological, psychological, and emotional changes, highly dependent on genetic, nutritional, and environmental factors. This time is traditionally divided in two main epochs, including the prenatal and the postnatal stage. The postnatal stage involves 5 phases, including infancy (neonate and up to 1 year age), toddler (1–2 years of age), childhood [early childhood (3–8 years), middle childhood (9–11 years)], adolescence (12–8 years), and adulthood (Cameron and Bogin, 2023).

From the prenatal stage to adulthood, cerebral networks undergo intensive development and continuous rearrangement, allowing for maturation of whole functional processing (Dennis and Thompson, 2013; Koenis et al., 2015; Cao et al., 2016; Meoded et al., 2017).

This process is strictly dependent on the correct building of white matter (WM) pathways, which form the substrate of cerebral structural connectivity. In fact, a large variety of WM tracts are needed to ensure a fast, efficient, multidistance (intralobar or loco-regional, extralobar intra-hemispheric, inter-hemispheric and extra-hemispheric) and multidirectional (intergyral, horizontal, vertical) integration between cortical and subcortical regions (De Benedictis and Duffau, 2011; Sarubbo et al., 2015, 2020). On the other hand, many diseases, especially observed during the developmental stage, may reflect different alterations in WM architecture.

Moreover, as demonstrated in adults, awareness of the network organization of the CNS may also be of special interest for the neurosurgical treatment of many diseases, improving the quality of surgical results while minimizing the risks of long-term post-operative morbidity (De Benedictis and Duffau, 2011; Duffau, 2021a). Although there are a flourishing number of neuroimaging studies focused on different aspects of WM development, there is no comprehensive vision linking this research to pediatric neurosurgical applications in the current literature.

Following the framework recently adopted for the cerebellar domain (De Benedictis et al., 2022), the aim of this review is to summarize the main current evidence on maturation dynamics of the supratentorial structural connectivity, to outline the functional correlations reported in both normal and pathological conditions, and to discuss the possible implications of this knowledge in neurosurgical practice.

## 2 The developing connectome

### 2.1 General perspective

#### 2.1.1 Exploring the developing connectome

The study of maturation dynamics, properties, and variabilities of the human structural brain network has seen impressive growth, thanks to continuous technological progress, data availability and methodological refinements. The earliest anatomical observations came from standard post-mortem studies on hematoxylin/eosin, Luxol fast blue, and immunohistochemical staining with the aim to assess chronological and topological myelination progression (Theodor, 1907; Flechsig, 1920; Yakovlev and Lecours, 1967; Brody et al., 1987; Kinney et al., 1988).

A revolutionary contribution to the exploration of WM anatomy came from Joseph Klingler (1888–1966), who in 1935 introduced an innovative method for the preparation of human specimens, allowing for the easier visualization and dissection of WM fascicles (Klinger, 1935; Agrawal et al., 2011; De Benedictis et al., 2018; Dziedzic et al., 2021). It is worth noting that, despite the initial dissemination and a currently renewed interest for this technique, almost no data has been collected on the application of Klingler dissection for the analysis of WM connections in the developing brain (Horgos et al., 2020). This is likely due to a lower feasibility of this technique, which is based on irreversible, progressively destructive, and macroscopic observation for the analysis of still highly fragile and immature WM structures.

The advent of non-invasive neuroimaging techniques, particularly MRI, made possible *in vivo* quantitative and qualitative characterization of the cerebral connectivity, opening the door to a new generation of exploration into connectome growth (Paus, 1998; Thompson et al., 2000; Paus et al., 2001; Dennis and Thompson, 2013; Dubois et al., 2014; Koenis et al., 2015; Mohammad and Nashaat, 2017; Lebel et al., 2019). In fact, as indicated by rapidly growing literature, consistent advancement has been achieved in both the application of established methods as well as in the development of new approaches to characterize the many aspects of cerebral connectivity, including the physiological spatio-temporal maturation process, the macro and microstructural properties of axons, the functional physiological and pathological developmental trajectories, and the influences of individual differences and external variables (Hermoye et al., 2006; Verhoeven et al., 2010; Deoni et al., 2012; Dean et al., 2015; Deshpande et al., 2015; Kulikova et al., 2015; Weiskopf et al., 2015; Zhao et al., 2015; Dubois et al., 2016; Krogsrud et al., 2016; Ouyang et al., 2017; Lebel and Deoni, 2018; Tamnes et al., 2018; Jin et al., 2019; Lebel et al., 2019).

Emerging research is now moving towards the development of other non-invasive methods beyond MRI, such as magnetoencephalography (MEG), electroencephalography (EEG), electrocardiography, near-infrared spectroscopy, cortico-cortical spectral responses, and cortico-cortical evoked potentials. As a result, modern “meta-connectomics” utilize these methods to produce a more exhaustive characterization of the developing brain network by integrating anatomical data (structural connectivity) with information on interneural interactions (functional connectivity), causal interdependencies (effective connectivity), directionality, and time, thereby creating a “6-dimensional connectivity” approach (Collin and van den Heuvel, 2013; Cao et al., 2016; Crossley et al., 2016; Sonoda et al., 2021).

#### 2.1.2 The structural connectome maturation

**Figure 1:**
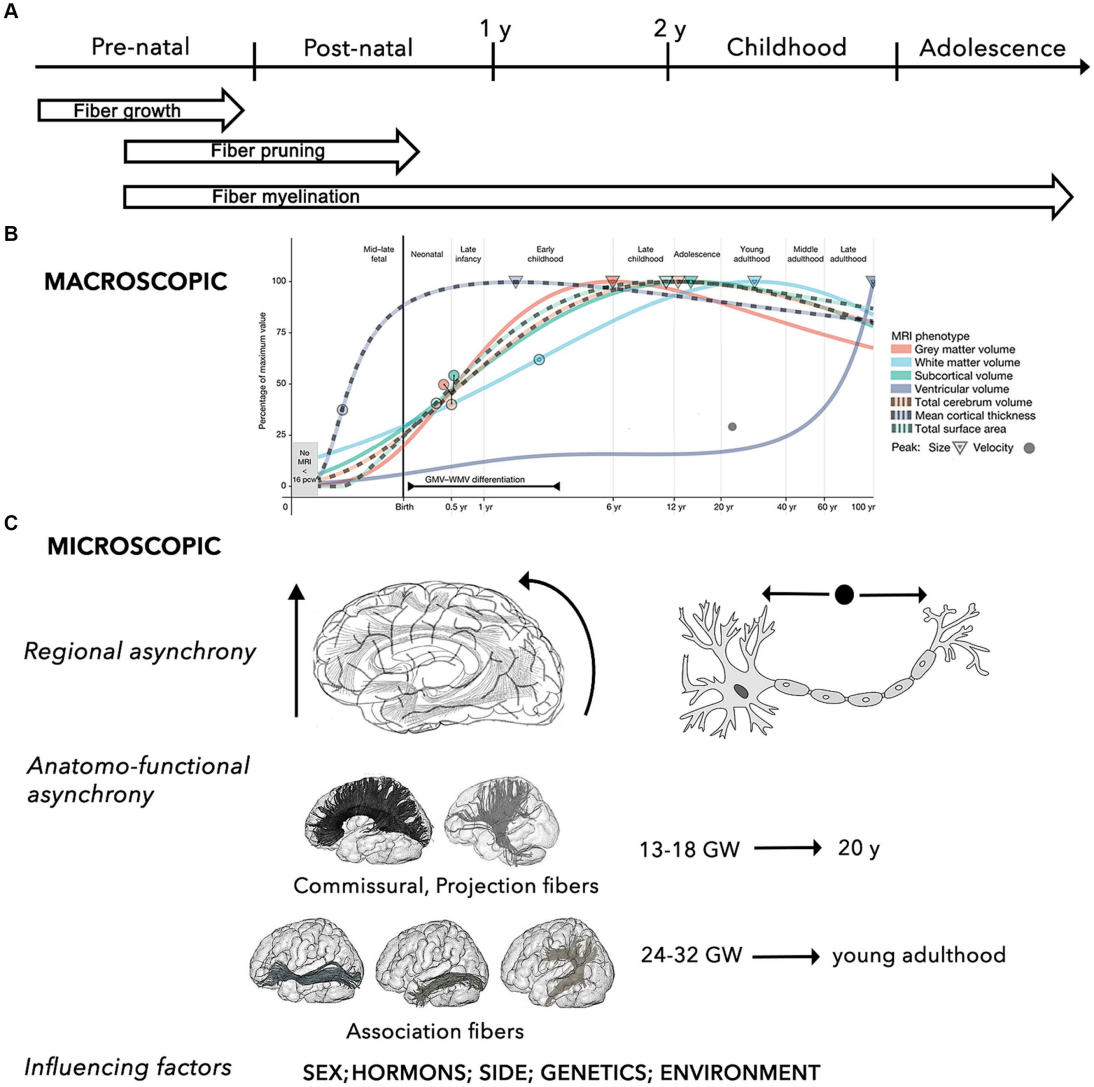
Summary of the key properties the brain structural network during the developmental process. **(A)** General time-line of mechanisms of WM maturation, including growth, pruning and myelination of axonal fibers during the pre-natal and post-natal periods. **(B)** Macroscopic developmental trends of the main brain MRI volumes (i.e., WM, gray matter, ventricles, subcortical area, cortical thickness, surface area, total cerebrum volume) across different stages of the human development. Circles and triangle indicate the peak rates of growth and volume for each trajectory, respectively (adapted from Bethlehem et al., 2022). **(C)** The micro-structural development is characterized by 3 main characteristics, including: (i) regional asynchrony, progressing from caudal to cranial, from posterior to anterior, and from the center to the periphery; (ii) maturation asynchrony of specific WM pathways, with commissural and projection connections developing earlier than association fibers, which progress into young adulthood; (iii) influence of different factors, such as sex and sex-related hormonal influences, side, and other environmental factors (e.g., prenatal alcohol exposure, anxiety, and depression, postnatal quality of caregiving, nutritional, and educational support) (Lebel and Deoni, 2018).

The development of the human brain network is a complex, long-lasting, and dynamic process (Figure 1). It starts during the third trimester of gestation and continues during infancy and adolescence, under the coordination of a myriad of molecular and cellular processes, regulated by individual, genetic and environmental factors (Huang et al., 2006, 2015; Lebel and Deoni, 2018). Thanks to progressive technological advancement, neuroimaging is currently able to detail an *in vivo*, quantitative, multi-parametric characterization of the spatio-temporal maturation of different WM bundles (Collin and van den Heuvel, 2013; Dennis and Thompson, 2014; Kulikova et al., 2015; Govaert et al., 2020).

Neuronal differentiation begins 4 weeks after fertilization. Between gestational week (GW) 12 and 20, neurons rapidly proliferate and migrate to different cortical sites under the guide of glial cells (Song et al., 2017). Neuronal migration leads to progressive formation of the primordial “preplate” (PP). Subsequent migrating neurons forming the “cortical plate” will split the PP in an outer layer, the “marginal zone,” and in an inner layer, the “subplate” (Govaert et al., 2020). Neuronal migration is followed by a period of apoptosis (i.e., programmed cell death), resulting in a massive reduction in the number of neurons. After reaching their cortical destination and until the end of the second trimester (GW 20-27/32), maturation of axons and dendrites forms the “intermediate zone” beneath the subplate, allowing for the development of inter-neuronal afferent and efferent connections (Govaert et al., 2020). This step is followed by a phase of selective pruning of connections and suppression of redundant or aberrant circuits, called fasciculation. The architecture of neuronal circuits is modeled by this process of continuous synaptogenesis, axonal growth, and pruning, resulting in corticofugal, thalamocortical, and commissural networks (Dubois et al., 2014; Yeatman et al., 2014; Govaert et al., 2020).

Maturation of the myelin sheath begins around GW 29 (Brody et al., 1987; Kinney et al., 1988; Dubois et al., 2014). While most of these critical events occur and advance more rapidly throughout the first 2 to 5 years of life, myelination continues through adolescence and adulthood, and undergoes continuous refinements throughout the lifespan, reaching its peak in the 2nd or 3rd decade of life (Yakovlev and Lecours, 1967; Lebel and Deoni, 2018).

The myelination process follows an asynchronous course, macroscopically progressing along a posterior-to-anterior topographical sequence, while following a proximal-to-distal progression at the microscopical axonal level (Paus, 1998; Paus et al., 2001; Prastawa et al., 2010; Sotardi et al., 2021). Hemispheric asymmetry has also been described, with earlier WM maturation in the left hemisphere for the frontal, temporal, and parietal regions, and in the right hemisphere for the thalamus, basal ganglia, and hippocampus (Sotardi et al., 2021).

Concerning the developmental patterns of specific pathways, several anatomical and neuroradiological studies based on analysis of fractional anisotropy (FA) and mean diffusivity (MD) [i.e., some of the most adopted parameters of WM structure and myelination, as well as cortical plate maturity] showed that, in general, all tracts are characterized by significant nonlinear modifications with age (Lebel and Beaulieu, 2011; Dubois et al., 2014). Specifically, maturation of commissural and projection fibers occurs earliest, emerging in the fetal brain between GW 13 and 18, while association connections mature at later ages (GW 24–32), and continue maturing after birth (Brouwer et al., 2012; Genc et al., 2018).

Using a longitudinal approach, it has been shown that, while gray matter volume progressively decreases, the volume of most WM tracts (especially projection and callosal fibers) increases significantly to become mostly complete by late adolescence (Eluvathingal et al., 2007; Lebel and Beaulieu, 2011; Nevalainen et al., 2014; Chen et al., 2016; Stephens et al., 2020; Bethlehem et al., 2022).

In addition to long-range connections, short-range connections are characterized by running parallel to the cortical surface, having incomplete myelination until the third decade of life, and maturing earlier and inconstantly in respect to the long association and callosal fibers, with a decreased growth from 2 to 16 years followed by increasing from 16 to 25 years (Ouyang et al., 2017).

As a result, the general macroscopic architecture of the brain connectome appears completed by the end of normal gestation (GW 37-42), showing an adult-like organization even if functionally immature (Flechsig, 1920; Hermoye et al., 2006; Dubois et al., 2008; van den Heuvel et al., 2015; Horgos et al., 2020). WM myelination and development of long-range connections are also strongly related to normal cortical maturation (Friedrichs-Maeder et al., 2017).

The subsequent post-natal course allows for further complex sharpening of the intrinsic microstructural architecture of brain networks, particularly during the first 2 years, by increasing integration, robustness, and efficiency of neural circuits, while decreasing local clustering and modularity. These modifications will progressively shift the intrinsic network microstructure from a random state to a more organized and stable configuration, until reaching to the typical “small-world” topology (Fair et al., 2009; Hagmann et al., 2010; Mishra et al., 2013; Cao et al., 2016; Khundrakpam et al., 2016).

The WM maturation process during childhood and adolescence is characterized by exponential, asynchronous, side-specific and sex-related changes. From a regional perspective, the intralobar connections within the frontal, parietal, and occipital lobes decreased with age, compared to an increased fiber density in the temporal lobe (Dennis et al., 2013). In parallel, more densely interconnected hubs were found at the level of the postero-medial core, the temporo-parietal junction, and the fronto-mesial cortices (Cao et al., 2016). Linear effects of increasing clustering, global efficiency, small-worldness, and modularity were found in the left hemisphere, with an opposite trend for the right hemisphere (Dennis et al., 2013).

Investigation on gender-related differences in WM maturation showed that the microstructural development occurs earlier in females, while it is more protracted in males (Lebel and Beaulieu, 2011). Moreover, the microstructural differences concerning specific territories and tracts, the related hormonal influences, and the possible implications for neurocognitive maturation have also ben hypothesized. For example, an MRI study of healthy children and adolescents (6–17 years) showed a significant right > left asymmetry in the total cerebral volume and in the WM volume of the middle and superior frontal gyri, and an age-dependent left > right increase of the inferior frontal gyrus volume in boys, speculating an association between these differences, and language development and lateralization (Blanton et al., 2004; Buyanova and Arsalidou, 2021).

Concerning maturation of specific WM pathways, a general pronounced increase in fiber density and myelination was observed in all major fiber tracts during transition from childhood to adolescence. However, the developmental trajectories are not linear, since maturation of projection and commissural tracts were found to be completed by late adolescence (20 years), whereas fronto-temporal and fronto-occipital association connections (i.e., superior longitudinal fascicle (SLF), inferior longitudinal fascicle (ILF), inferior fronto-occipital fascicle (IFOF), uncinate fascicle (UF), cingulum) showed a more protracted maturation cycle into young adulthood (Lebel and Beaulieu, 2011; Khundrakpam et al., 2016; Buyanova and Arsalidou, 2021).

Emerging observations from anatomical, functional, genetic, and graph-theory studies are providing evidence of a genetic influence on the shaping of brain networks during adolescence, allowing for a certain degree of heritability of WM integrity (e.g., within the left frontal lobe, the callosal splenium, and the right inferior longitudinal fasciculus), and the global and local efficiency of information transfer. Finally, despite limited and often controversial data, the investigation on other possible factors influencing individual differences showed that positive environmental influences, such as breastfeeding and proper nutrition, support WM development differently from negative influences, including prenatal exposures or early deprivation (Lebel and Deoni, 2018).

### 2.2 Structural networks and neurocognitive maturation

The earliest neuroanatomical and neurophysiological studies showed that the myelination process marks a breakthrough in the development process of brain circuits, by increasing the velocity of electrical conduction that allows for progressively improving the efficiency of interneural inhibitory and executive interactions throughout infancy, childhood, and adolescence and into adulthood (Buyanova and Arsalidou, 2021). More recently, the combined application of neuroimaging technology and sophisticated computational modeling of structural brain topology provided adjunctive evidence on the fact that a child’s connectome maturation is associated with a growing capacity of the brain network to integrate and reinforce functional information between its different subparts (Dosenbach et al., 2007; Fair et al., 2009; van den Heuvel et al., 2015). Moreover, computation of probabilistic maps allows for further detection of possible correlations between regional microstructural development of WM tracts and neuro-cognitive properties, skills, and abilities beginning in the early infant lifetime (Akazawa et al., 2016; Ouyang et al., 2017).

As for structural maturation, neurocognitive development also follows an asynchronous, age-specific timeline, with basic functions (sensory-motor, visual, auditory) developing earlier in respect to higher cognitive and emotional properties (Nagy et al., 2004; Pujol et al., 2006; Dean et al., 2015; Dai et al., 2019). It is likely that these chronological differences reflect a hierarchical organization, in which more primitive, localized, and dependent pathways might stabilize processing at higher-level of functions that require more mature, distributed, and interconnected systems (Guillery, 2005; Huang et al., 2006; Dubois et al., 2008; Kulikova et al., 2015).

Moreover, the WM microstructural development appears to be an adaptive phenomenon, involving fiber tracts that connect brain areas associated with various cognitive, affective, and motor functions, and susceptible to environmental influences and changes induced by learning, intense activity, or new experiences.

Different possible mechanisms have been described, including activity-dependent (i.e., dependent on electrical activity of the axon and various molecular mediators released in response to electrical events) and activity-independent new myelination phenomena or myelin remodeling of already myelinated axons (Tau and Peterson, 2010; Zatorre et al., 2012; Fields, 2015; Lebel and Deoni, 2018; Bells et al., 2019; de Faria et al., 2019; Buyanova and Arsalidou, 2021).

In the following section, we will summarize the development of specific networks, namely the sensory-motor, auditory and visuo-spatial, language, and intellectual.

#### 2.2.1 Sensory-motor network

The development of the sensory and motor systems begins between GW 12-18 and continues through the first 2 months of post-term life. Post-mortem studies in human infants showed that during the early preterm period (GW 26-34), thalamo-cortical axons grow from the subplate zone to the cortical plate and form a highly integrated system with cortico-thalamic connections, constituting the anatomical pathway for sensory impulses from the periphery to the cortex (Nevalainen et al., 2014). Maturation thalamo-cortical afferent projections first involves the internal capsule and the cerebral peduncles, and then progresses along an inferior–superior axis forming the corona radiata according to chronological order of the myelination process (Hermoye et al., 2006; Huang et al., 2006; Weinstein et al., 2014, 2016).

By GW 20, cortico-spinal motor projections sprout from the pyramidal corticospinal motor neurons, located in layer V of the sensorimotor area, and grow in a cortico-fugal direction to reach the alpha-motor neurons of the spinal cord and, to a lesser extent, to reach the striatum and brainstem nuclei (Eyre et al., 2000; Staudt, 2010). At the end of the embryonic period, the pyramidal tract reaches the level of the pyramidal decussation (GW 17) and proceeds towards the rest of the spinal cord, until lower thoracic cord (GW 19) and the lumbosacral cord (GW 29). Myelination of the pyramidal tract usually starts at the end of the second or the beginning of the third trimester and proceeds following a cranial-to-caudal direction, over a protracted period until 2-3 years of post-natal life (ten Donkelaar et al., 2004).

It has been demonstrated that myelination is an adaptive process, in which oligodendrocyte’s precursor cells would play a crucial role by modifying the structure of WM to improve motor learning and acquisition of new motor skills (McKenzie et al., 2014).

The following normal development is characterized by competition between ipsilateral and contralateral projections, with gradual weakening of ipsilateral fibers and strengthening of contralateral fibers (Eyre et al., 2001). The typical following post-term development is characterized by the emergence and disappearance of various patterns of motor sequences and regulation of the muscular tone, to provide a progressive adaptation to requirements of extra-uterine environment (Einspieler et al., 2008).

#### 2.2.2 Auditory and visuo-spatial network

Maturation of auditory perception and discrimination precedes that of the visual perception system (Weinstein et al., 2014). At the beginning of third trimester of pregnancy (from GW 25-27), myelination starts from the cochlear outlet through the brainstem and progressively develops involving the trapezoid body, the lateral lemniscus, the brainstem commissures, and the axons running from the inferior colliculus to the medial geniculate body (Moore and Linthicum, 2007). This corresponds to the early appearance of certain motor and behavioral responses to sound, until maturation by term age of fine auditory discrimination between expected and unexpected sounds (Stipdonk et al., 2016).

At GW 33-34, the capacity to discriminate and show differential visual preference to appropriate temporal and spatial stimuli is already present (Dubowitz et al., 1980; Geva et al., 1999; Hunnius et al., 2008). The neuro-biological model of typical early development of visuo-spatial processing during the first year of life depends on a complex and integrated system, which links analysis of visual inputs to visuo-motor control, visual cognition, and attention. The volume of primary visual cortex reaches an adult size by the age of 4 months, while synaptic density reaches adult levels after the age of 5 years (Huttenlocher et al., 1982). Although myelination of the optic radiation (OR) is completed by the age of 3 years, its microstructure maturation continues until adolescence (Kinney et al., 1988; Dayan et al., 2015).

The networks involve neurons of the striate cortex in the occipital lobe specialized to extract local visual information, and a series of extra-striate areas within the lateral occipital, temporal, parietal, and frontal lobes. These regions are integrated by two systems: (a) a ventral pathway, involving the ILF, specialized for recognizing shapes and objects, including human faces and colors; and (b) a dorsal pathway, involving the SLF and the arcuate fasciculus (AF) within the right non-dominant hemisphere, which encodes for spatial and motion information needed for visually guided actions (Atkinson and Braddick, 2020).

The progressive maturation of these circuits allows for development of specific functions, including cortical selectivity, integration of local signals to provide global representations of motion, shape and space, development of visuo-motor modules for eye movements, manual reaching, and locomotion, and development of distinct attentional systems (Geva et al., 1999; Hunnius et al., 2008; Harel et al., 2011).

#### 2.2.3 Language network

The anatomo-functional organization of language is one of the most debated neuroscientific topics over the last two centuries. Recent advances in functional neuroimaging techniques and intraoperative electrical mapping have allowed our understanding to move past the traditional localizationist framework (i.e., Broca’s area = language production; Wernicke’s area = language comprehension), in favor of a more dynamic interplay between large-scale cortico-subcortical sub-networks (David et al., 2011; Friederici et al., 2011; Duffau et al., 2014; Sarubbo et al., 2016; Duffau, 2018; Debenedictis et al., 2021). Currently, the most accepted model describes a dual route system, in which input information is processed and transmitted from temporo-parieto-occipital regions to frontal output articulatory motor programs through a dorsal phonological and syntactic stream and a ventral semantic pathway, all under the strict executive control of “amodal” deeper circuits (Duffau et al., 2014; Skeide and Friederici, 2016).

According to this view, maturation of language abilities is strictly related to development of a complex cortical and subcortical network (Brauer et al., 2011; Rosselli et al., 2014; Skeide and Friederici, 2016). It was demonstrated that, at the earliest period of language acquisition when infants begin to vocalize (6 to 22 weeks of life), the macroscopic organization of WM pathways is analogous to adults in terms of axonal microstructure and fiber trajectories (Dubois et al., 2016).

On the other hand, children’s network organization differs from that of adults in two main aspects. First, it has been shown that normal processing of auditory, phonemic, and prosodic inputs in newborns depends on bilateral involvement of frontal and temporal cortices. This is due to a stronger inter-hemispheric connectivity in newborns than in adults of, mainly due to the corpus callosum (CC), which develops by 12 weeks of post-natal age (Feng et al., 2019). Callosal connections are responsible for coordination and integration between the two hemispheres. By the age of 2 years callosal connections contribute to inter-hemispheric functional lateralization, leading to dominance of the left hemisphere for segmental information (phonemes, syllables, morphemes, and words) and dominance of the right hemisphere for prosodic processing (Perani et al., 2011).

Second, there are more local sensorimotor, auditory, and visual networks than distributed long-range networks observed in children than are observed in adults. Subsequent language acquisition depends on the progressive development of intra-hemispheric association connections, which starts from the 13th postnatal week (Dubois et al., 2014). The dorsal and ventral pathways are already clearly segregated at the early developmental stage and constitute the anatomical foundation for an efficient interaction between posterior and anterior brain regions. As for phylogenetic development, maturation of ventral and dorsal streams is asynchronous, with ventral tracts (i.e., IFOF and ILF) already present at birth, while dorsal bundles (i.e., AF and SLF) mature later. This maturation gap is overcome during the first weeks of post-term life (Friederici et al., 2011; Dubois et al., 2016).

Moreover, two different maturation patterns have been identified for the dorsal stream. Fibers terminating in the pre-motor cortex appear during newborn infancy and are involved in the early phase of auditory-motor integration during language learning in infancy (Friederici, 2012). On the other side, fibers reaching the dorsal inferior frontal gyrus, develop at age of 7 years and are involved in more complex linguistic aspects (Perani et al., 2011; Brauer et al., 2013; Mohades et al., 2015; Wilkinson et al., 2017).

An interesting approach is the analysis of structural covariance, describing the phenomenon that gray matter properties and axonal connectivity of one brain area may co-vary with those of other distributed cortical regions (Geng et al., 2017). The resulting functional interaction between regions depends on a combination of factors including mutually trophic influences, age, shared experience, and behavior related plasticity. Concerning language, the structural covariance of cortical thickness between the left frontal and the left temporal regions was shown to be positively related to sentence comprehension abilities in preschool children, whereas a stronger association between the gray matter structural covariance and the WM connectivity of the same homologous regions, was found in adults in relation to syntactic abilities (Qi et al., 2019).

Finally, an innovative approach based on a multi-connectivity analysis (“6-dimensional tractography”) has been recently reported to investigate properties and trajectories of effective connectivity of language networks in the developing brain, by integrating and visualizing into a single model disparate information about network structure, functional interactions, strength, causal relationships, directionality, and time. Using this approach, based on the evaluation of the cortico-cortical responses during electrocorticography recording in more than 2000 temporal and extratemporal sites, a more robust connectivity between the temporal lobe and extratemporal regions during the response preparation period compared to listening and overt response, was found in older subjects, with a preferential quantitative involvement of the AF according to a temporal-to-extratemporal direction (Sonoda et al., 2021).

#### 2.2.4 Intellectual abilities networks

Several studies assessing the impact of WM properties on higher intellectual processing in children demonstrated significant correlations between WM development and inter-network integration for a large range of abilities, including cognitive, affective, motivational, social behavior, visual–spatial reasoning, sensorimotor integration, spatial problem activities, response inhibition, executive function, response time variability, working memory, mathematical performance and reasoning, mnemonic control, letter-number sequencing, and sustained attention (Parks and Madden, 2013; Deoni et al., 2016; Wendelken et al., 2017; Tamnes et al., 2018; Girault et al., 2019; Goddings et al., 2021).

Development of intellectual performance progresses according to an age-dependent process of microstructural maturation and myelination of association and projection pathways, which sustain the cortical and brainstem integration across childhood and adolescence into adulthood. Interaction between biological mechanisms and experiential learning leads to progressively configure different specialization patterns, to improve reaction time, and to increase the transfer efficiency of local information throughout the different hubs of the network (Peters et al., 2014; Kim et al., 2016; Buyanova and Arsalidou, 2021).

Maturation of critical cognitive functions is associated with a region-specific increase of axonal volume and myelination of several WM tracts [such as the SLF, the IFOF, the ILF, the cingulum and the corpus callosum (CC)] (Peters et al., 2014). For example, auditory working memory processing will be sustained by a network involving the parietal cortical areas, the left superior and posterior corona radiata, and the body of the CC. Executive functions will develop according to maturation of fronto-parietal networks. Maintenance of visuospatial attention will be associated with myelination of the right optic radiation, the right posterior thalamus, and the right medial precuneus. Myelination of the left superior corona radiata and the left ILF will correlate to numerical operations and mathematical reasoning. Maturation of CC will mediate the interhemispheric signal transduction, especially between the right parietal cortex to the left inferior parietal cortex, for both inhibitory control and executive functions at the base of object recognition processing (Buyanova and Arsalidou, 2021).

It is worth noting that a direct involvement of age was not constantly demonstrated. For example, for reading and working memory, analysis of diffusion maps revealed that a significant correlation between WM and FA for left inferior frontal, left occipito-temporal, and CC clusters remained even after removing the effect of age. This indicates that efficiency of neurocognitive development might depend not only on factors related to maturation, such as myelination or axonal diameter growth, but also on the quality of fiber organization within the network (Nagy et al., 2004).

## 3 Developing network and pathology

**Figure 2:**
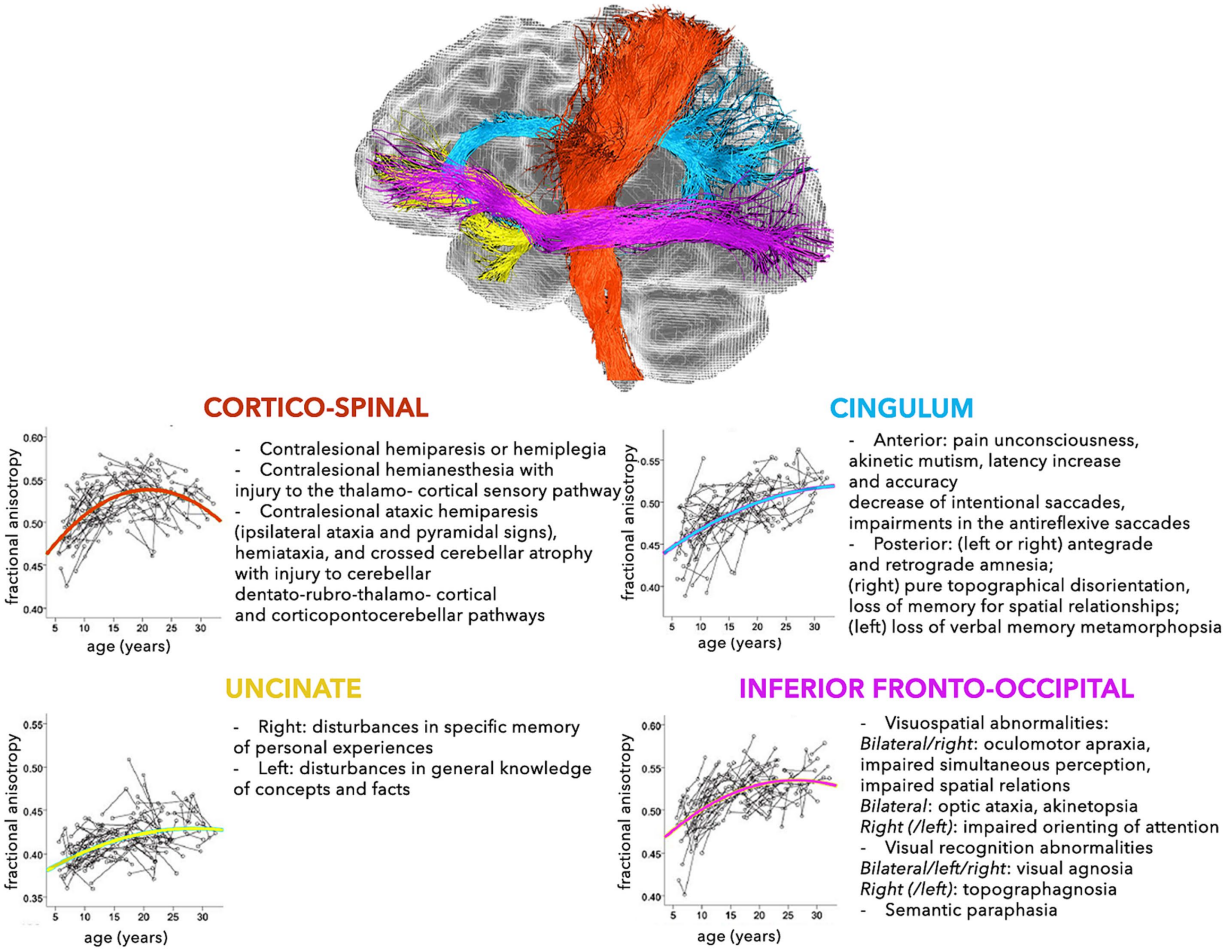
Schematic representation of the normal and pathological trajectories of the main WM pathways (part 1). For each tract, the longitudinal age-related changes of fractional anisotropy (modified from Lebel and Beaulieu, 2011) (*left*) and the possible pathological manifestations (*right*) are shown (Aralasmak et al., 2006).

**Figure 3:**
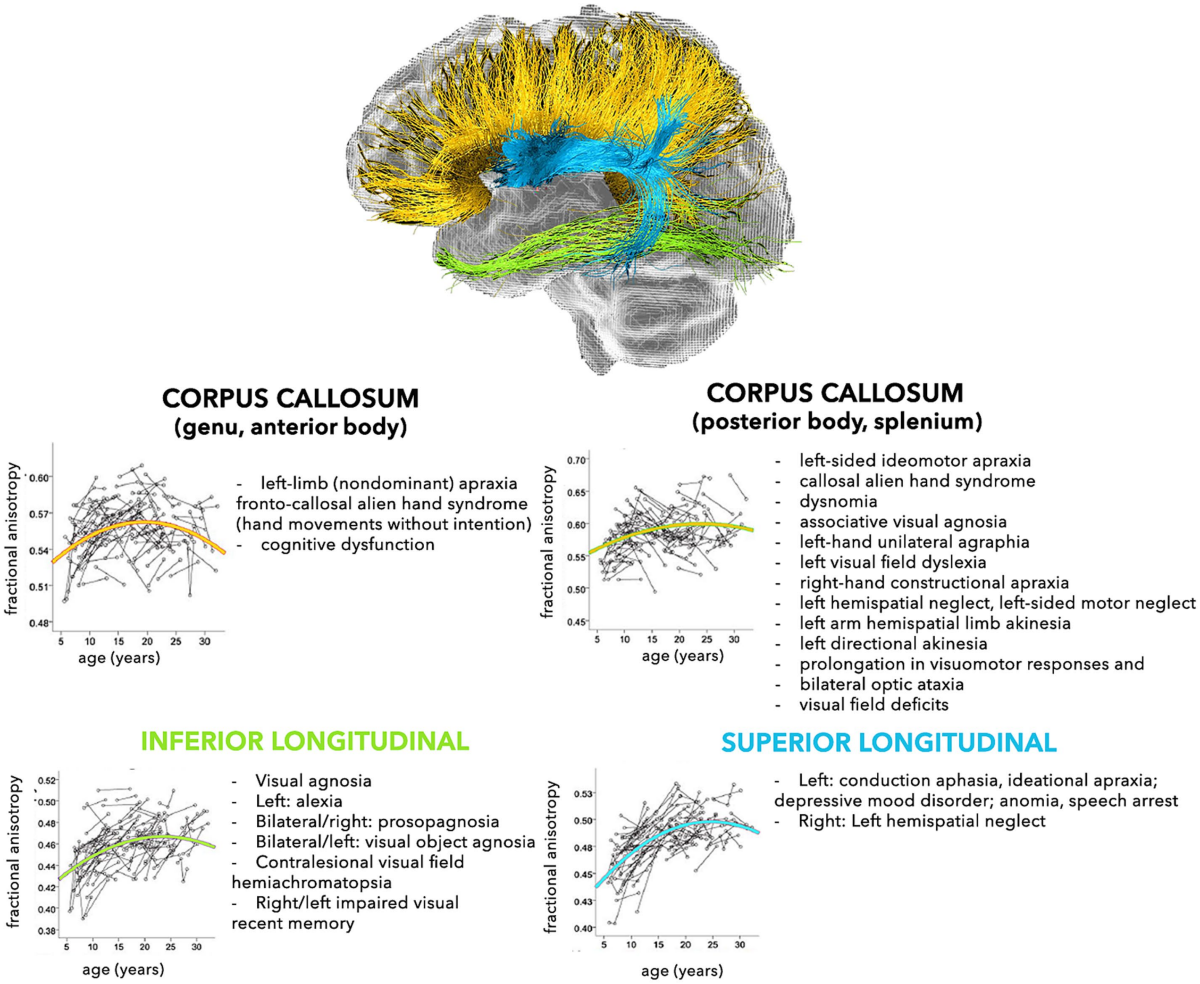
Schematic representation of the normal and pathological trajectories of the main WM pathways (part 2). For each tract, the longitudinal age-related changes of fractional anisotropy (modified from Lebel and Beaulieu, 2011) (*left*) and the possible pathological manifestations (*right*) are shown (Aralasmak et al., 2006).

The modern network-based approach to the anatomo-functional organization of the CNS is corroborating the hypothesis that several disorders may depend on deviation from normal configuration of brain networks towards pathological patterns (Alstott et al., 2009; Collin and van den Heuvel, 2013). This is especially true for younger subjects, who are more vulnerable, due to low number, robustness, and stability of brain connections (Huang et al., 2006, 2015). In fact, a large spectrum of pathological processes involving the WM, such as genetic, demyelinating, infectious, inflammatory, toxic, metabolic, vascular, traumatic, malformation, and neoplastic diseases are reported in childhood (Habes et al., 2022). The neurological consequences may depend on the specific WM tracts involved (Figures 2, 3), but also on possible repercussions on the whole CNS architecture (Aralasmak et al., 2006; Sharp et al., 2014).

The mechanisms leading to post-lesion plastic reorganization depend on the different possible complexities of brain circuit configuration. In fact, motor and sensory systems are more primitive, have a more localized topography, and have relatively few WM pathways. Contrastingly, cognitive systems develop later and continue to evolve long after birth. Moreover, these systems are more distributed and interconnected, potentially permitting a more extensive intra-hemispheric and inter-hemispheric reorganization (Anderson et al., 2011; Stiles et al., 2015). For this reason, there is increasing interest in adopting connectomic biomarkers for predicting neurodevelopmental outcomes in infants with pre-natal or perinatal diseases (Girault et al., 2019).

In the following section the main aspects affecting the impact of specific pathological situations on WM architecture will be summarized. According to the aim of this review, we will focus on the diseases with the most neurosurgical interest.

### 3.1 Stroke

Cerebro-vascular events may produce different neurological deficits, related to the size, topography, and timing of the ischemic lesion. In general, focal ischemic lesions primarily affect circumscribed brain regions and fiber tracts resulting in acute neurological deficits. At a later stage, apoptosis, inflammation, neurodegeneration, and diaschisis phenomena can also affect remote areas of the brain, resulting in a more widespread secondary perturbation of more widely distributed networks, even in the non-ischemic hemisphere (Thiel and Vahdat, 2015).

In preterm infants (20 week of gestation – 28^th^ day of postnatal life), one of the most frequent cause of focal stroke is intraventricular hemorrhage (IVH). This condition, resulting from hemorrhage, infarction, and/or edema, may cause direct disruptions of the periventricular WM, causing to axonal loss and poor myelination. The most affected tracts are the optic radiations, the cingulum, the anterior limb of the internal capsule, the fornix, the SLF, the ILF, the IFOF, and the CC. The entity of WM alterations has been found to be directly correlated with gestational age at birth, and severity of IVH has been found (Kirton et al., 2021; Triplett and Smyser, 2022).

These factors reflect impairment at stroke onset, but are also correlated with potential clinical recovery, depending on specific mechanisms of post-lesional reorganization of the afferent and efferent pathways involved. Four main phases characterize the brain repair processes, including cellular lysis and inflammation, diaschisis, secondary death, expansion, and focalization. These phenomena, promoted by learning or relearning allow for synaptic inputs regulated by hyper- and hypo-excitability, modification, and redistribution of synaptic strength, allowing for development of interneural connections and synchronization or inhibition of aberrant non-functional circuits (Loubinoux et al., 2017).

Concerning the sensory-motor system, lesions involving any part of the cortico-spinal tract (CST), namely at the level of the cortex, the basal ganglia, and the posterior limb of the internal capsule, almost are always predictive of a motor deficit after stroke (Husson et al., 2010). In the early stage of normal development, CST projections to the spinal cord are bilateral. The following maturation process consists in activity-dependent or trophic-like interactions between the two halves of the CST, according to four key-mechanisms, including synaptic competition between the CSTs from each hemisphere, interactions between the CST and spinal cord neurons, synaptic competition between the CST and proprioceptive sensory fibers, and interactions between the developing corticospinal motor system and the rubrospinal tract.

The transient existence of ipsilateral corticospinal projections followed by preferential contralateral and unilateral evolution of fibers provides the base for both normal motor development and post-lesional reorganization. For this reason, consistent relationships between alterations in the WM connectome of the non-lesioned hemisphere and clinical function were demonstrated (Williams et al., 2017).

Recent studies, based on the reconstruction of structural connectivity by tractography, combined with mathematical models using graph theory approach, allowed to elucidate the atypical connectivity of the non-lesioned hemisphere, the correlation with motor disability, and the potential neuroplastic reorganization of these areas after perinatal stroke (Yu et al., 2018; Craig et al., 2020, 2022).

In particular, using transcranial magnetic stimulation and diffusion tensor imaging (DTI), three patterns of motor reorganization have been identified in a series of both pediatric and adult patients, including (i) ipsilateral control of the impaired hand by the intact hemisphere, by persistent ipsilateral CST projections, (ii) involvement of the contralateral CST by the damaged hemisphere, and (iii) combined ipsilateral and contralateral CST control of the impaired hand. The type of reorganization of the CST depends on several factors including the type and timing of the injury, the size and location of lesion, and the plasticity potential of the CST itself (Staudt et al., 2002, 2004). At a microscopic level, the site of primary lesion is characterized by the higher degree of altered myelination with progressive normalization towards remote ipsilesional and contralesional regions (Mackay et al., 2023).

Therefore, the gravity of post-stroke motor and other neurological deficits often correlates with the lesion size, and especially during the early third trimester, with involvement of the medial periventricular WM, where cortico-spinal motor pathways run from the primary motor cortex to the internal capsule (Dinomais et al., 2015; Stiles et al., 2015). Moreover, if a prenatal or perinatal arterial ischemic stroke occurs unilaterally before or during the time of synaptogenesis of corticospinal motor projections (up to the age of 2 years), the ipsilateral fibers from the hemisphere contralateral to the lesion persist into maturity and replace the activity of damaged projections (Staudt et al., 2002, 2004; Staudt et al., 2008; Staudt, 2010).

The clinical entity of an ischemic involving the somato-sensory system event mainly depends on the corresponding development stage of afferent projections to the postcentral cortex. If a periventricular lesion occurs before the normal maturation of the thalamo-cortical connections (i.e., third trimester of pregnancy), the thalamo-cortical somatosensory projections can bypass the lesion site to reach anyway the cortical destination (Staudt et al., 2008). Contrasting the motor system, it has been demonstrated that reorganization of sensory afferents lacks cortical plasticity and tends to remain contralateral regardless of the time of the injury, without shifting from the damaged to undamaged hemisphere (Staudt et al., 2008).

Concerning language, early neonatal stroke has a higher potential of anatomo-functional reorganization due to a more flexible language network in comparison with lesions acquired in a highly specialized functional network during adulthood (Staudt et al., 2001, 2002; François et al., 2019). In fact, the analysis of functional and structural connectivity in pre-school children with left perinatal arterial ischemic stroke indicates that lesions of the left dorsal pathway and the associated perisylvian regions may induce the degeneration of neurons projecting from temporal to frontal areas while triggering the interhemispheric transfer of language functions to right homologous regions, allowing for positive language outcomes (François et al., 2019).

However, despite the high plastic potential of infant brain, it seems that an early injury to the left dorsal language cannot be compensated by the ventral stream and that, in case of absence of interhemispheric transfer, long lasting speech repetition problems, like conduction aphasia seen in adults, may persist (Northam et al., 2018).

Finally, evaluation of the impact of age (1 month and 5 years of age, and between 6 and 16 years of age) at time of stroke on higher neurocognitive abilities in children with a history of unilateral stroke during the perinatal period, revealed that, although earlier age at stroke was associated with weaker cognitive performance overall, the relationship between age at injury and cognitive outcome is non-linear and modulated by other variables such as the specific cognitive skill, lesion type, and lesion location. Strokes involving subcortical structures were found to be most detrimental to intellectual ability and information processing skills when occurring during the prenatal or early perinatal period (before the age of 28 days), probably because the higher vulnerability of early developing networks that ins associated with a less efficient compensation during maturation of higher-level and later-developing cognitive skills (Chapman et al., 2003; Westmacott et al., 2010).

### 3.2 Trauma

Traumatic brain injury (TBI) is an important cause of death and disability in the pediatric population (Königs et al., 2017). Growing evidence supports the role of diffusion MRI (dMRI) as a sensitive diagnostic tool to characterize different patterns of WM alterations in patients with TBI (Imms et al., 2019).

The acute post-traumatic phase is characterized by increasing of FA and decreasing of MD, likely reflecting oedema and inflammation, with a reversing trend during the sub-acute and chronic phases of TBI, probably due to wallerian degeneration, or to a slower rate of myelin development (Barzó et al., 1997; Marmarou et al., 2006; Wilde et al., 2012; Yallampalli et al., 2013). However, these measures can be quite heterogeneous across TBI patients, depending on type, location, and severity of the injury, pre-existing conditions, home support, and other factors (Dennis et al., 2015). A direct correlation between the Glasgow coma scale and WM integrity was also debated with discrepant result across different groups (Yuan et al., 2007; Dennis et al., 2015).

Several authors have analyzed the post-TBI WM damage by both focusing on different brain regions and specific WM tracts (Xu et al., 2007; Bendlin et al., 2008; Wu et al., 2011; Pal et al., 2012; Wilde et al., 2012; Bartnik-Olson et al., 2021).

In pediatric subjects, neurocognitive and behavior impairments would depend on post-traumatic widespread axonal damage within the developing brain of both animal model and human subjects (Babikian and Asarnow, 2009; Li and Liu, 2013; Königs et al., 2017). Different patterns of abnormal connectivity have been found in children older than 7 years, depending on the post-traumatic temporal interval in children following moderate/severe TBI. Post-acutely (1-5 months post-injury), less significant differences have been detected between TBI patients and controls. In the chronic phase of recovery (13-19 months post-injury), more significant consequences were found in the structural connectome, including increased path length, indicative of reduced structural integration with poorer neurocognitive outcome, especially in intelligence and working memory domain (Dennis et al., 2015; Königs et al., 2017).

Despite a considerable amount of inter-subject heterogeneity, the body, genu, and splenium of the CC, the IFOF, the ILF, the anterior thalamic radiation, the fronto-striatal circuit, and the cingulum were the most reported structures involved (Wu et al., 2011; Dennis et al., 2015; Königs et al., 2017). Consequently, TBI may produce a “disconnection syndrome,” where symptoms would depend on an altered local and global connectivity, so that a damage occurring within one area may also involve other distant but connected brain regions, according to the diaschisis phenomenon (Feeney and Baron, 1986).

More recently, application of graph theoretical analysis to TBI revealed that normalized clustering coefficient and characteristic path length can be adopted to characterize the post-traumatic shift from a normal network configuration and to predict consequent functional alterations (Park and Friston, 1979; Babikian and Asarnow, 2009; Li and Liu, 2013; Sharp et al., 2014; Yuan et al., 2015). According to this approach, a poorer post-TBI neurocognitive outcome has been demonstrated especially in chronic TBI patients of all severity types, and in acute mild TBI patients with persistent symptoms (Kraus et al., 2007; Kinnunen et al., 2011).

Quantification of network structural connectivity based on graph theory and DTI tractography has emerged as a promising tool to investigate also the post-traumatic modifications of structural connectivity, in response to cognitive training and intervention, and to characterize the association between these changes and neurobehavioral outcomes in children after the initial injury (e.g., improvements in verbal working memory associated with a decreased MD in the left SLF) (Caeyenberghs et al., 2012; Li et al., 2014; Yuan et al., 2017; Imms et al., 2019; Verhelst et al., 2019). On the other side, as demonstrated by animal DTI and histopathological studies, post-injury rearrangement of cerebral connectivity may determine maladaptive effects on neural processing at the basis of long-term physical, cognitive, psychological, and emotional impairments, and adversely influence the recovery and rehabilitation process in survivors of pediatric TBI (Li et al., 2014).

### 3.3 Hydrocephalus

Hydrocephalus is a very common disease in childhood. Despite different possible etiologies and still debated pathophysiological mechanisms, hydrocephalus depends on imbalances between production and absorption of the CSF, with consequent enlargement of the cerebral ventricles and increasing of intracranial pressure (Williams et al., 2015; Isaacs et al., 2019). Even more advanced diffusion MR imaging techniques have allowed researchers to assess the integrity of periventricular WM, and to correlate the microstructural effects related to hydrocephalus with neurodevelopmental outcomes. D-MRI alterations have been demonstrated also in asymptomatic chronic or mild hydrocephalus cases, and in patients without ventricular dilatation, supporting the concept that dMRI may be a sensitive measure of WM injury, independently from clinical symptoms of increased intracranial pressure and ventricular sizes (Isaacs et al., 2019). Findings in animal models and in humans have demonstrated that pathological ventricular expansion produces different types of brain injury, including gradual degradation and demyelination of periventricular WM, as well as hypoxic insults to oligodendrocytes due to post-compression reduction of cerebral blood flow (Khan et al., 2006; del Bigio, 2010; Olopade et al., 2012).

In specific situations, such as post-hemorrhagic hydrocephalus, there is also evidence that intraventricular hemorrhage induces a secondary WM injury through iron-mediated free radicals and hypoxanthine-derived oxidative damage (Cherian et al., 2004; Isaacs et al., 2019). The most vulnerable structures to these effects are the CC, the internal capsule, the fornix, as well as the periventricular projection axons, leading to neuromotor and cognitive developmental deficits seen in children (Isaacs et al., 2019).

Studies demonstrated that hydrocephalus-related WM impairments may be reversed by shunt surgery and that WM fibers that undergo axonal damage may have a worse and slower recovery following treatment than those that undergo altered or delayed myelination (Air et al., 2010; Isaacs et al., 2019). Consequently, diffusion parameters have been suggested as suitable biomarkers for evaluating renormalization of WM tracts, in parallel with the reduction in ventricular size, during the long-term follow-up after CSF shunting or revision surgery and to correlate these data with the clinical outcome and quality of life (Tan et al., 2018).

More recently, analysis of the correlation between executive function impairments at school age (6-10 years) in children with infantile HCP and brain network structural and functional connectivity revealed patterns of both hypo-connectivity and hyper-connectivity, especially between the ventral attention network and fronto-parietal and dorsal attention networks, between the limbic and fronto-parietal network. These alterations might reflect not only the long-term interactions between evolving large-scale networks, but also would also represent compensatory effects within areas of the same networks in response to white matter damage after HCP (Adam et al., 2022).

### 3.4 Epilepsy

Epilepsy is one of the most common diseases occurring during childhood, with a wide spectrum of possible etiologies, seizure semiologies, electrophysiological patterns, and neuroradiological features (Minardi et al., 2019). Growing evidence is now overcoming the traditional concept of a region-specific origin of epilepsy, while supporting the implication of an altered anatomo-functional organization of neural circuits, that is an epileptogenic network formation, in which seizures would generate and propagate (Chavez et al., 2010; Zhang et al., 2011; Richardson, 2012; Engel et al., 2013; Taylor et al., 2014; Xue et al., 2014; de Palma et al., 2020). Studies based on brain diffusion demonstrated that childhood epilepsy may be associated and induce several types of developmental WM abnormalities, including disruption of axonal integrity, alteration of fiber organization and density, myelin abnormalities, and delayed maturation, with a consequent disconnection among different brain regions that leads in turn to further disruption of cortical and subcortical connectivity (Hutchinson et al., 2010; Widjaja et al., 2013; Ciumas et al., 2014).

A relationship between epilepsy onset and specific patterns of WM damage was also found, with generalized tonic–clonic attack typically associated with bilateral tract weakening, and focal seizure onset in case of unilateral WM impairment (Evstigneev et al., 2013). On the other side, it has been demonstrated that epilepsy itself may induce modifications of the cerebral WM architecture, with a consequent disconnection among different brain regions that leads in turn to further disruption of cortical and subcortical connectivity (Bhardwaj et al., 2010).

The result is an impaired global efficiency of the functional information transfer that would be responsible also for neurocognitive disorders frequently seen in children with new-onset seizures, particularly in the domains of intelligence, language, psychomotor speed, and executive function (Widjaja et al., 2013; Ciumas et al., 2014; Kim et al., 2014; Paldino et al., 2014, 2017; Parker et al., 2018).

Distinct patterns of WM abnormalities have been investigated across different epilepsy subtypes (Slinger et al., 2016; Pujar et al., 2017).

In general, WM alterations may occur at the very earliest phases of epilepsy. For example, microstructural abnormalities, especially in the posterior CC and the cingulum, were identified in patients with both localization related and idiopathic generalized epilepsies with recent onset (12 years) and short duration of epilepsy (less than 12 months), without other developmental disabilities or neurological disorders. These patterns may indicate a preferential damage to later myelinating callosal regions or that the myelination of axons in the patients is slowed by the epileptogenic process (Hutchinson et al., 2010).

In focal epilepsy, it has been shown that WM changes are significantly associated with younger age at epilepsy onset, longer epilepsy duration, and male sex (Slinger et al., 2016). These diffusion modifications are usually not restricted to the ipsilateral hemisphere but may also involve areas remote from the suspected lesion, with severity patterns depending on different types of focal epilepsy (Govindan et al., 2008).

Temporal lobe epilepsy (TLE) and mesial temporal lobe epilepsy (mTLE) are the most frequent forms of focal drug-resistant epilepsy and the most studied models of epileptogenic network (Imamura et al., 2016). In patient with TLE due to different etiologies, different histological anomalies have been identified in relation with WM alterations that would be also correlated with epilepsy duration and sensitivity to medical treatment (Glenn et al., 2016). Moreover, damage of WM structure has been identified not only within the temporal region, but also in distant regions, with possible widespread involvement of connected structures, through a large network of different associations (IFOF, ILF, SLF, UF, cingulum), projections (anterior and posterior internal capsule), and commissural (anterior and posterior CC) bundles, either unilaterally or bilaterally (Powell et al., 2007; Yogarajah and Duncan, 2007; Concha et al., 2010; Günbey et al., 2011; Otte et al., 2012).

Concerning pathophysiology underlying the bilateral changes in WM structure observed in TLE patients, two theories have been proposed (Imamura et al., 2016; Roger et al., 2018). The first theory indicates that diffusion anomalies to the contralateral epileptogenic hemisphere are symmetrical and secondary to a direct primary effect of the epileptic activity propagation. The second theory states that WM alterations would be initiated by a direct effect of the local epilepsy network, followed by progressively centrifugal and decreasing changes of WM tracts through several possible mechanisms. These include secondary wallerian degeneration due to repetitive seizure spread, hypoxia and vasoconstriction, antiepileptic drug treatment, altered brain development, and plasticity-related reorganization of local and global circuits (Concha et al., 2010; Günbey et al., 2011; Otte et al., 2012; Pujar et al., 2017).

TLE epilepsy is considered a paradigmatic model for the investigation of the impact of epilepsy-related plastic phenomena on language and memory performance.

In fact, the complementary application of both a functional and structural approaches allow to estimate the functional lateralization of linguistic capacities in TLE patients, in relation to structural asymmetry of language-related tracts (namely, the AF, UF, and ILF), and to reveal possible subcortical regions of increased connectivity that could reflect the inter-hemispheric and intra-hemispheric functional compensation (Roger et al., 2018).

Alterations of microscopic WM integrity would also be related to cognitive comorbidity in patients with chronic epilepsy with a temporal or fronto-temporal focus (McDonald et al., 2008; Riley et al., 2010; Vaessen et al., 2012).

In mTLE, WM abnormalities are usually associated with hippocampal sclerosis (Kreilkamp et al., 2021). In these cases, especially chronic and severe cases, alterations of WM tracts running through the temporal lobe, namely the UF and the ILF, have been found. Moreover, decreased axonal density of the fimbria and involvement of the fornix bilaterally or unilaterally, and of other more distant extratemporal or contralateral structures have been observed in both adults and children, indicating the role of persistent seizures in recruiting various neuroanatomic structures composing a seizure network (Kimiwada et al., 2006; Shon et al., 2010; Liu et al., 2013; Xu et al., 2014).

Epilepsy with an extratemporal origin, such as in children with a frontal lobe epilepsy is usually associated with cognitive impairment. These patients exhibited selective action-concept deficits associated with structural and functional abnormalities along motor networks (cortico-spinal tract, anterior thalamic radiation, uncinate fasciculus) (Holt et al., 2011; Braakman et al., 2014; Moguilner et al., 2021). Moreover, WM abnormalities have been found in posterior brain regions, beyond the area of the seizure focus, confirming that the seizure rapid propagation from the frontal lobe may influence the WM maturation process. Interestingly, these modifications were not associated with any of the clinical epilepsy characteristics considered, especially age at epilepsy onset, seizure frequency, localization of seizure focus, and antiepileptic drug use (Braakman et al., 2014).

In case of epilepsy due to brain malformations, such as lissencephaly, nodular periventricular heterotopia, schizencephaly, hemimegalencephaly, focal cortical dysplasia (FCD), association between structural networks alterations and myelination timing has been also identified (Arrigoni et al., 2020; Subramanian et al., 2020).

In fact, in one case of early postnatal epilepsy due to hemimegalencephaly, myelination development was accelerated in the cerebral hemisphere in which seizures occurred in respect to the normal contralateral hemisphere, suggesting that neuronal activity, even if abnormal, positively modulates the process of myelination in the neighborhood of the epileptogenic focus as well as along functionally linked WM tracts (Goldsberry et al., 2011).

Moreover, it has been shown that the timing of insult during corticogenesis impacts the extent and severity of topological network anomalies and of consequent structure–function decoupling (Widjaja et al., 2007; Subramanian et al., 2020). For example, malformations occurring at earlier maturation stage may selectively interfere with formation of large-scale cortico-cortical networks, leading to a more severe impact on the whole-brain organization (Widjaja et al., 2007; Subramanian et al., 2020). On the contrary, malformation occurring at later stage of cortical development, such as FCD type II, tends to affect WM more locally at the level of fibers underlying the dysplastic cortex (Hong et al., 2017).

Nevertheless, recent studies reported that FCDs may be associated with widespread abnormalities of structural connectivity beyond or distal to the lesion, or even regardless of the specific FCD location (Eriksson et al., 2001; Gross et al., 2005; Widjaja et al., 2007, 2009; de Carvalho et al., 2012; Rezayev et al., 2018; Mito et al., 2022). For example, patients with epilepsy associated with bottom-of-sulcus dysplasias (BOSDs) show a widespread, bilateral, and symmetrical reduction of structural connectivity independent on the lesion laterality, involving different projection, association, interhemispheric, and cerebellothalamic tracts. Three main, not-exclusive mechanisms have been hypothesized to explain the association between connectivity abnormalities and epilepsy related to BOSDs. These include direct causal interdependence, complete independence, or indirect influence between both abnormal structural networks (Mito et al., 2022).

In patients with non-lesional epilepsy and genetic predisposition to cortical hyperexcitability and delayed brain maturation, alteration of neuronal structural connectivity appears to be not necessarily related to duration of the disease but may be also established prior to the seizure onset (Holmes et al., 2001; Ciumas et al., 2014; Slinger et al., 2016; Kreilkamp et al., 2021).

In generalized epilepsy WM changes have been found to be less pronounced than in focal epilepsy. Several WM regions of altered diffusion patterns, especially concerning projection and callosal fibers, have been identified in previous series concerning all subjects with epilepsy onset during childhood (Slinger et al., 2016).

For example, recent studies in animal models revealed that absence seizures, (i.e., a generalized seizure type characterized by behavioral arrest that occurs in multiple forms of generalized epilepsy), can induce activity-dependent myelination, which in turn promotes further progression of epilepsy, according to the concept of maladaptive myelination.

The aberrant myelination changes, such as increase in myelin sheath thickness and in myelinated axons, are extensive, but network-specific, with preferential involvement of the corpus callosum and the anterior commissure (Knowles et al., 2022; Chau Loo Kung et al., 2023).

In patients with idiopathic generalized epilepsy both microscopic and macroscopic abnormalities in thalamo-cortical and cortical–cortical pathways have been demonstrated using diffusional kurtosis imaging. These two patterns would have different implication in epileptogenesis, with macroscopic volumetric changes being directly associated with seizure activity, while microstructural abnormalities would represent subtle neurodevelopmental network rearrangements occurring before the epilepsy onset (Zhang et al., 2011; Lee et al., 2014).

In juvenile myoclonic epilepsy DTI and probabilistic tractography studies revealed a significant reduction of microstructural and macrostructural connectivity of fronto-striatal networks, involving the supplementary motor area, the putamen bilaterally, and thalamocortical WM. Also in this case, these architectural alterations would be significantly related to early age of onset and a longer seizure history (Keller et al., 2011; Vulliemoz et al., 2011).

### 3.5 Tumors

Tumors can affect the brain structural connectivity in many ways, depending on the specific histopathology and natural history, including by infiltrating, interrupting, shifting WM pathways, increasing local pressure, and inducing cerebral hypoperfusion (Raybaud, 2016). Growing studies indicated that a tumor may interfere with the whole CNS architecture, going beyond a strictly topographic criterium (Ailion et al., 2017; Özyurt et al., 2017).

It is worth noting that the current literature devoted to characterizing brain connectome alterations in the context of supratentorial brain tumors in childhood is relatively limited (Ostrom et al., 2015; Ailion et al., 2017). The available studies mainly concern lesions harboring the posterior fossa, which is the most common location of pediatric brain tumors (Rueckriegel et al., 2015; McEvoy et al., 2016; Udaka and Packer, 2018; De Benedictis et al., 2022).

Moreover, the effect of local WM damage on larger scale structural networks has not been clearly explored. This might depend on the different tumor biology and natural history between childhood and adulthood. In fact, children have a higher incidence of well-demarcated, not infiltrating, and indolent forms of low-grade gliomas than adults (Trevisi et al., 2016). Adult lesions are characterized by continuous growth, infiltration of eloquent cortical and subcortical territories and anaplastic transformation (Trevisi et al., 2016).

Most studies evaluate the effects after treatment, especially radiotherapy, while only few studies have examined the direct effect of tumors on the WM microstructure and their consequent neurologic and neurocognitive sequelae (Reddick et al., 2003; Stavinoha et al., 2018; Peterson et al., 2019).

In general, concerning pediatric supratentorial lesions, it has been shown that brain regions characterized by a later development and myelination are more susceptible to microstructural damage and, consequently, to impairment of neurocognitive performance (Reddick et al., 2003; Wier et al., 2019). However, in patients with a history of low-grade tumors treated without radiation therapy it has been shown that the lesion can be associated with and even predict the possible effects on cognitive, social, and emotional performance (Peterson et al., 2019).

These aspects may be not exclusively attributable to specific tumor-related characteristics (type, size, location) and comorbid medical aspects, but are associated with different possible patterns of brain network impairment, including direct cortical damage, WM disconnection at local or remote level, or both (Bartolomei et al., 2006; Honey and Sporns, 2008; De Benedictis and Duffau, 2011; Özyurt et al., 2017).

Finally, a lesion localized in the same region may induce different symptoms, depending on the individual variability of the cortical and subcortical organization of the specific sub-network, and on the possible anatomo-functional post-lesion reshaping (Ghazwani et al., 2021; Duffau, 2022).

Four levels of plasticity have been identified in the adult population, with a hierarchical order of the compensatory recruitment, involving first intra-lesional and peri-lesional regions, and later contra-lesional territories. The main factors influencing these dynamics and the consequent functional implications include the rapidity of the lesion growth, usually low in pediatric tumors, and the involvement of the subcortical connectivity that plays a major role in promoting the development of compensatory networks. Therefore, well-circumscribed tumors observed in pediatric tumors allow a higher degree of plasticity due to the relative sparing of WM tracts, in comparison with more diffuse and infiltrating gliomas observed in adults (Cargnelutti et al., 2020; Nieberlein et al., 2023).

In fact, DTI studies and results coming from intraoperative DES showed that plastic potentials of WM are not unlimited and may represent a constraint in case of tumor invasion. Among the different WM structures, a gradient of inter-system and intra-system plasticity have been described, with low compensation capacity for the cortico-spinal tract, except for the most dorsal-anterior portion, and greater potentials for WM fascicles involved in language and, among these, more essential role of the ventral stream (namely the IFOF), in respect to the dorsal way (i.e., the AF).

Recently, a core of essential, non-compensable, and therefore non-resectable brain structures have been identified, especially in areas of the networks having no parallel alternative pathways (Ius et al., 2011; Sarubbo et al., 2020).

## 4 Neurosurgical implications

An accurate knowledge of the anatomo-functional properties and developmental dynamics of children’s brain connectomes may also have crucial implications for the neurosurgical community aiming to achieve the best outcome while minimizing risks of post-surgical long-term deficits.

Although the most consistent body of literature concerns adults, growing studies are now being devoted to the pediatric population. Studies showed that surgery is responsible itself for alterations on the WM architecture that may have potential consequences on neurocognitive outcomes (Stavinoha et al., 2018). Moreover, even if plastic potentials are especially expressed during the brain maturation process, the complexity of subcortical circuitry and the lower capacity of post-injury restoration attributed to WM structures in respect to the cortical domain is directly related to the need for careful awareness of the short-term and long-term neurological consequences of surgery (Delion et al., 2015).

Consequently, development and systematic application of accurate non-invasive preoperative planning and neuromonitoring is of critical importance for preserving the main components of the network (Rosenstock et al., 2020). However, unlike adult neurosurgery, in which neurophysiological techniques during surgery in eloquent areas are well established, accuracy of intraoperative monitoring results is still limited for pediatric cases, mainly due to the immaturity of nervous structures and more limited study of younger neurocognitively compromised patients (Trevisi et al., 2016; Alotaibi et al., 2021).

Following these considerations, in the next section we discuss aspects linking pediatric brain connectivity with current and possibly future perspectives of neurosurgical management, particularly regarding the pediatric experience on epilepsy and neuro-oncological surgery.

### 4.1 Epilepsy surgery

In parallel with the conceptualization of epilepsy as a network disease, growing studies are investigating possible implications of structural brain connectivity in the surgical approach to epilepsy. To this regard, some aspects are of special interest and will be mentioned here. These include (i) the relevance of knowledge of WM anatomy in surgical management, (ii) the possible effect of surgery on structural connectivity of an epileptic brain, (iii) the influence of epilepsy as a network pathology on surgical results, and (iv) the implication of brain connectivity for the selection of the most effective surgical approaches.

The two main categories of procedures include resections and disconnections, depending on the location and size of the target, defined on the base of seizure semiology, electrophysiological, and MRI data assessed during the presurgical evaluation (Guan et al., 2016; Dallas et al., 2020). In both these situations, an accurate awareness of anatomy of WM pathways is crucial, even with the paradoxically opposite goals of preserving eloquent WM connectivity during resections in eloquent areas, and achieving a complete separation between lobar, multi-lobar, or hemispheric territories and the rest of the brain in case of disconnection procedures.

In this context, increasing studies reported the application of standard post-mortem dissection for representing the relevant WM surgical anatomy related to different approaches for epilepsy (Kucukyuruk et al., 1982, 2012; Verhaeghe et al., 2018; Flores-Justa et al., 2019). Moreover, tractography reconstructions based on DTI or diffusion weighted imaging (DWI) have been proposed as useful tools to plan safer approaches in regions close to eloquent pathways, to verify postoperatively the completeness of a disconnection, or to correlate postsurgical neurological deficits with WM impairments (Radhakrishnan et al., 2011; Lee et al., 2013; James et al., 2015; Jeong et al., 2015; Küpper et al., 2016; Binding et al., 2022).

Over the last decade, progressively more advanced techniques are allowing researchers to evaluate the possible effects of surgery on the WM connectome in children operated on for drug-resistant epilepsy. For example, a postoperative increase of contralateral axonal connectivity (anisotropy may involve both myelination, and number of axonal fibers) has been identified for different pathways, including the OR after resections involving the temporal, parietal, and occipital lobes; the whole intra-hemispheric connectivity of the unaffected hemisphere after hemispherotomy procedures; the insulo-fronto-opercular, and superior and mid fronto-orbital connections in a pediatric series of patients after undergoing temporal and extra-temporal resections; and after a case of surgeries involving the left AF (Govindan et al., 2008; Goradia et al., 2011; Ibrahim et al., 2015; Jeong et al., 2016; Li et al., 2018; Lacerda et al., 2020). These modifications are strongly suggestive of the occurrence of an adaptive or compensatory reorganization of the structural connectivity beyond the age-related maturational changes, presumably triggered by the surgery itself (Govindan et al., 2008; Skirrow et al., 2015; Jeong et al., 2016).

Some authors have evaluated whether the analysis of the brain connectome might be related to and even predictive of the quality of postoperative outcome. There is much evidence supporting the role of early childhood neurosurgery in inhibiting the progression of WM fiber disruptions induced by epilepsy, supporting earlier seizure control and better neurocognitive development (Slinger et al., 2016; Mito et al., 2022). In hemispherotomy cases, studies indicated the role of tractography in helping identify sites of possible incomplete disconnection in seizure-recurrent patients, or in predicting motor function outcome based on the asymmetry of the cortico-spinal tract volume within the brainstem (Toda et al., 2014; Küpper et al., 2016). Comparison between the preoperative and postoperative structural connectivity in temporal resection cases revealed distinct reorganization patterns in local and distal networks associated with postsurgical seizure freedom or recurrence (Goradia et al., 2011; Ji et al., 2015).

Finally, the framework of epilepsy as a network disease is encouraging the investigation on the possibility of adopting the structural connectivity as a reliable criterium for selecting the most appropriate and safe surgical strategy and technique, including alternative approaches to standard resections and disconnections. For example, in case of focal cortical lesions located within deep or eloquent territories, some Authors indicated the utility of direct electro-stimulation during stereo-EEG monitoring to differentiate the pathological WM connections from the normal connectome, with the aim of identifying and possibly treating through minimally invasive procedures the aberrant components of the epileptic network, without damaging the normal structures (Bourdillon et al., 2019). Concerning periventricular nodular heterotopias, the development of SEEG-guided radiofrequency procedures allowed to both characterize and ablate the significant epileptic network associated to different components of the malformation with high rate of responders (Bourdillon et al., 2018). Regarding hypothalamic hamartomas (HHs), many studies demonstrated a strong association between different altered brain networks, mainly involving the thalamus, the temporal and frontal regions and the HH-related epileptogenesis, and the often-associated endocrinological and neuropsychological disorders. This lead to develop more focused approaches alternative to classical open resections, (i.e., including endoscopic disconnection, radiosurgery and stereotactic thermo-ablation or laser-ablation), with the aim of interrupting the pathological network by deafferenting the HH from the thalamus and the mammillary bodies (Bourdillon et al., 2021).

More recently, Giampiccolo et al. (2023) performed a retrospective analysis of the patterns of disconnection associated with long-term seizure outcome in a series of 47 patients who underwent surgery for frontal lobe epilepsy. The Authors found a significant correlation between seizure freedom and disconnection of the anterior thalamic radiation and the cortico-striatal tract, indicating the role of these pathways in the development of novel epileptic networks at the base of seizure recurrence. Therefore, they recommended to extend the surgical approach beyond the resection of the target area, also including the disconnection of WM networks recognized as involved in seizure generation and propagation.

It is worth noting that data on this topic are quite promising, yet relatively new and still limited to the adult population, while current studies in children concern analysis of functional connectivity. Although different methodologies were used, the available data seems to confirm that the surgically related changes of children’s functional network are conversely related to the epilepsy outcome (Iandolo et al., 2021).

### 4.2 Neuro-oncological surgery

Currently, surgery is considered the first-choice treatment for most of brain tumors occurring during childhood, and gross total resection is the most consistent prognostic factor to achieve prolonged progression-free status (Greuter et al., 2021; Thorbinson and Kilday, 2021). However, evidence of possible late motor and non-motor deficits among survivors allowed researchers to investigate the possible effects of surgery and adjuvant treatments (chemotherapy and radiotherapy) on WM integrity and, consequently, on physiological development and neurological functioning, especially in younger patients (Wolfe et al., 2012; Robinson et al., 2013; Stavinoha et al., 2018). Concerning the neurocognitive outcome, it has been established that children treated for brain tumors may be influenced by several variables associated with different treatment modalities (Stavinoha et al., 2018).

Beyond the well documented effects of cranial radiation, the cognitive effects of surgery on brain connectivity have received less attention in the literature (Nagesh et al., 2008). The most significant studies reported that alterations of WM structures were predictive of adverse reductions in intellectual function independent from the tumor location (Liu et al., 2015; Peterson et al., 2019; Aleksonis et al., 2021). These reductions include verbal working memory, brief attention/vigilance, rapid psychomotor output, and visual perception and matching, and weaknesses after resection of pediatric brain tumors without radiotherapy (Liu et al., 2015; Peterson et al., 2019; Aleksonis et al., 2021).

Other possible implications in more specific contexts have been investigated. Significant correlation between postoperative impairment of neurocognitive performance and WM alterations, especially of the middle frontal gyrus, has been demonstrated in a series of 15 children after resection of lateral ventricular low-grade tumors through a frontal transcortical approach (Zhu et al., 2017).

Following authors have evaluated the possible advantage on neurocognitive outcomes of an alternative approach (i.e., the transcallosal route) in respect to the transcortical corridor for resecting tumors located within the same region. Interestingly, while preoperative and postoperative variations were significant for some cognitive domain deficiencies related to the specific approach, no significant difference was found when comparing the two surgical routes (He et al., 2020). In patients who underwent surgery for craniopharyngioma, DTI was used to assess the risk of neurotoxicity associated with proton therapy, confirming that regions that developed WM defects due to a surgical approach may be more susceptible to radiation dose-effects (Uh et al., 2015).

As important consequences of these observations, careful preoperative planning, intraoperative neuronavigation, and neurophysiological monitoring are crucial also in pediatric neurosurgery, especially in case of high-grade and low-grade tumors located within eloquent regions (de Oliveira et al., 2015; Coppola et al., 2016; Celtikci et al., 2017; Foley and Boop, 2017; Foster et al., 2018; Abdel Razek et al., 2020).

In addition to conventional tractography and neuronavigation techniques, some innovative solutions have recently been suggested to better enhance the relationships between tumors and WM pathways, optimizing the surgical performance and the neurological outcome. For example, strengthening reconstruction methods of specific tracts, such as the optic radiation, has been proposed to improve the pediatric neurosurgical outcomes (Yang et al., 2019). A combined use of whole brain tractography, neuronavigation, tubular retraction, and exoscope visualization has been reported for approaching a pediatric left thalamic/midbrain pilocytic astrocytoma (Weiner and Placantonakis, 2017). Association of trans-magnetic stimulation and DTI data have been described to visualize the spatial relationships among tumor, motor cortex, and motor and language tracts in a pediatric series, with good feasibility, accuracy of non-invasive functional mapping, parent counseling, and neurological outcome (Rosenstock et al., 2020). Finally, although still lacking in applications in pediatric neurosurgery, advances in imaging and computer technology are leading to the development of advanced software for three-dimensional modeling, sophisticated virtual and augmented reality simulators, to improve the surgeon’s understanding of spatial relations of anatomical landmarks, to select the best surgical approaches, and to verify intraoperative performance (Rehder et al., 2016; Kin et al., 2017; Cho et al., 2020).

Intra-operative neuromonitoring (ION) is considered as the “gold standard” method to identify and preserve the critical structures at both the cortical and the subcortical levels and to validate results of presurgical planning, so optimizing both the surgical and functional outcomes (Coppola et al., 2016). Mostly derived from studies on adults, growing literature now demonstrates an increasing application of ION in pediatric practice over the last decade, not only when pathology involves the posterior fossa, the brainstem, or the spinal cord region, but also when involving the supratentorial compartment.

In a recent series of 57 cases in which ION was used for motor mapping and monitoring, the authors found significant correlation between cortical and subcortical threshold and immediate transitory postsurgical motor decline. Moreover, neurophysiological evocability was effective by the age of 2 years, while it appeared to be related neither to tumor location nor to the pathological grade (Roth et al., 2020). Concerning other intraoperative mapping techniques, direct electrical stimulation in awake conditions is currently a well-established and largely adopted method in adult neurosurgical practice, especially for monitoring language and higher neurocognitive domains (Sarubbo et al., 2015; Ferracci and Duffau, 2018; Herbet and Duffau, 2020; Sarubbo et al., 2020; Duffau, 2021b; Herbet, 2021).

For pediatric procedures, growing series and technical notes have been recently reported (Delion et al., 2015; Trevisi et al., 2016; Lohkamp et al., 2019; Alcaraz García-Tejedor et al., 2020; Lohkamp et al., 2020; Ratha et al., 2021; Herta et al., 2022). In the last available review, results of 18 studies concerning 50 patients who underwent awake surgeries for tumors, epilepsy, and functional diseases are reported. Although different indications and procedures were reviewed, the authors confirm the safety, feasibility, and well-tolerability of awake procedures, with gross total resection achieved in 16 out 18 reported cases (88%), and post-operative permanent deficits overall observed in 4% of patients. The main “state-of-the-art” recommendations proposed by the authors include accurate pre-operative interdisciplinary planning, an optimal technical quality for intraoperative electrostimulation, neuronavigation, and neuromonitoring, as well as the essential role of a careful neuropsychological support during presurgical preparation, the intraoperative phase, and the follow-up (Lohkamp et al., 2019).

## 5 Conclusions and future perspectives

Modern neuroscience has significantly progressed towards a more realistic anatomo-functional characterization of the CNS, definitively surpassing the dogma of a strict and presumptive correlation between cerebral topography and function, in favor of a largely integrated and plastic network. Connectomics has emerged as a convincing paradigm for exploring the complex modeling and the continuous refinement of the intra- and inter-hemispheric WM connections, highlighting the normal neurocognitive development, and the possible pathophysiological mechanisms at the base of many brain disorders.

This evidence should not be limited to theoretical speculation, as it has important implications for pediatric neurosurgical practice, especially for the management of brain tumors and selected epilepsy cases. In fact, the application of multimodal neuroradiological and neurophysiological approaches would enable a multidisciplinary team to tailor the pre-operative assessment as well as the intra-surgical and post-operative management to the specific network configuration of each patient.

However, many aspects are relatively unexplored or under-documented in the pediatric context, for both research and clinical purposes. Some could be mentioned as still open topics and interesting possible subjects for future investigation.

These concern, for example: (i) methodology for the assessment of brain networks, effects of the pathological process, and cerebral plasticity, based on a qualitative and quantitative longitudinal and multimodal approach; (ii) the accuracy of neurophysiological techniques, especially for the intraoperative cortical and subcortical monitoring, especially of higher-level functions, such as language. To this regard, the application of emerging methods, such as cortico-cortical evoked potentials, in patients that cannot undergo awake procedures, will extend the feasibility of intraoperative monitoring of language circuits also to the pediatric population (Yamao et al., 1977; Matsumoto et al., 2004; Giampiccolo et al., 2021); (iii) the application of machine learning (ML) technology, based on the identification of specific structural WM biomarkers for the computation of reliable probabilistic atlases based on different convergent factors, such as the presurgical lesion characteristics, the relationships between the lesion type and the local and global network, the functional interactions and outflows, and the possible clinical deficits; (iv) the development of model-based approaches, based on computation of brain network models by integrating data from reference atlases with anatomo-functional connectivity data coming from patient-specific imaging, and electrophysiological recordings (e.g., EEG patterns identified by stereo-EEG recordings). As already described in both the adult and pediatric epilepsy literature, the combined use of brain network model representation (the so-called “virtual epilepsy brain models”) with ML and artificial intelligence methods would allow to approximate the extent and the organization of the epileptogenic zone (EZ), intended as the site of beginning and of the primary organization of epileptic seizures. This information might contribute to simulate the consequences of different hypothetical EZs, to stratify the postoperative risk, to simulate surgeries, to predict the postsurgical outcome, to improve the quality of communication to patients and families, with the ultimate goal of optimizing surgical management, clinical outcome, and neurorehabilitation strategies (Bonilha et al., 2015; Sinha et al., 2017; An et al., 2019; Jirsa et al., 2023).

We believe that these may represent challenging yet promising foundations for building a reliable, personalized, and safe “connectome-based” pediatric neurosurgery.

## Author contributions

AB and MR-E contributed to conception and design of the study. AB wrote the first draft of the manuscript. AB, MR-E, and LP wrote sections of the manuscript. All authors contributed to the article and approved the submitted version.

## References

[ref1] Abdel Razek A. A. K. El-Serougy L. Ezzat A. Eldawoody H. El-Morsy A. (2020). Interobserver agreement of White matter tract involvement in gliomas with diffusion tensor Tractography. J. Neurol. Surg. A Cent. Eur. Neurosurg. 81, 233–237. doi: 10.1055/s-0039-1700560, PMID: 31777049

[ref2] Adam R. Ghahari D. Morton J. B. Eagleson R. De Ribaupierre S. (2022). Brain network connectivity and executive function in children with infantile hydrocephalus. Brain Connect. 12, 784–798. doi: 10.1089/brain.2021.0149, PMID: 35302386

[ref3] Agrawal A. Kapfhammer J. P. Kress A. Wichers H. Deep A. Feindel W.. (2011). Josef Klingler’s models of white matter tracts: influences on neuroanatomy, neurosurgery, and neuroimaging. Neurosurgery 69, 238–252. doi: 10.1227/NEU.0b013e318214ab79 21368687

[ref4] Ailion A. S. Hortman K. King T. Z. (2017). Childhood brain tumors: A systematic review of the structural neuroimaging literature. Neuropsychol. Rev. 27, 220–244. doi: 10.1007/s11065-017-9352-6 28646252

[ref5] Air E. L. Yuan W. Holland S. K. Jones B. Bierbrauer K. Altaye M.. (2010). Longitudinal comparison of pre- and postoperative diffusion tensor imaging parameters in young children with hydrocephalus. J. Neurosurg. Pediatr. 5, 385–391. doi: 10.3171/2009.11.PEDS09343 20367345

[ref6] Akazawa K. Chang L. Yamakawa R. Hayama S. Buchthal S. Alicata D.. (2016). Probabilistic maps of the white matter tracts with known associated functions on the neonatal brain atlas: application to evaluate longitudinal developmental trajectories in term-born and preterm-born infants. NeuroImage 128, 167–179. doi: 10.1016/j.neuroimage.2015.12.026, PMID: 26712341 PMC4762721

[ref7] Alcaraz García-Tejedor G. Echániz G. Strantzas S. Jalloh I. Rutka J. Drake J.. (2020). Feasibility of awake craniotomy in the pediatric population. Paediatr. Anaesth. 30, 480–489. doi: 10.1111/pan.13833 31997512

[ref8] Aleksonis H. A. Wier R. Pearson M. M. Cannistraci C. J. Anderson A. W. Kuttesch J. F.. (2021). Associations among diffusion tensor imaging and neurocognitive function in survivors of pediatric brain tumor: a pilot study. Appl. Neuropsychol. Child 10, 111–122. doi: 10.1080/21622965.2019.1613993, PMID: 31146596

[ref9] Alotaibi F. Mir A. Al-Faraidy M. Jallul T. Al-Baradie R. (2021). Pediatric awake epilepsy surgery: intraoperative language mapping utilizing digital video gaming and electrocorticography. Epilepsy Behav. Rep. 17:100521. doi: 10.1016/j.ebr.2021.100521 35118367 PMC8792417

[ref10] Alstott J. Breakspear M. Hagmann P. Cammoun L. Sporns O. (2009). Modeling the impact of lesions in the human brain. PLoS Comput. Biol. 5:e1000408. doi: 10.1371/journal.pcbi.1000408, PMID: 19521503 PMC2688028

[ref11] An S. Bartolomei F. Guye M. Jirsa V. (2019). Optimization of surgical intervention outside the epileptogenic zone in the virtual epileptic patient (VEP). PLoS Comput. Biol. 15:e1007051. doi: 10.1371/journal.pcbi.1007051, PMID: 31242177 PMC6594587

[ref12] Anderson V. Spencer-Smith M. Wood A. (2011). Do children really recover better? Neurobehavioural plasticity after early brain insult. Brain 134, 2197–2221. doi: 10.1093/brain/awr103, PMID: 21784775

[ref13] Aralasmak A. Ulmer J. L. Kocak M. Salvan C. V. Hillis A. E. Yousem D. M. (2006). Association, commissural, and projection pathways and their functional deficit reported in literature. J. Comput. Assist. Tomogr. 30, 695–715. doi: 10.1097/01.rct.0000226397.43235.8b 16954916

[ref14] Arrigoni F. Peruzzo D. Mandelstam S. Amorosino G. Redaelli D. Romaniello R.. (2020). Characterizingwhite matter tract organization in polymicrogyria and lissencephaly: a multifiber diffusion mri modeling and tractography study. Am. J. Neuroradiol. 41, 1495–1502. doi: 10.3174/ajnr.A6646, PMID: 32732266 PMC7658898

[ref15] Atkinson J. Braddick O. (2020). Visual development. Handb. Clin. Neurol. 173, 121–142. doi: 10.1016/B978-0-444-64150-2.00013-7 32958168

[ref16] Babikian T. Asarnow R. (2009). Neurocognitive outcomes and recovery after pediatric TBI: meta-analytic review of the literature. Neuropsychology 23, 283–296. doi: 10.1037/a0015268, PMID: 19413443 PMC4064005

[ref17] Bartnik-Olson B. Holshouser B. Ghosh N. Oyoyo U. E. Nichols J. G. Pivonka-Jones J.. (2021). Evolving White matter injury following pediatric traumatic brain injury. J. Neurotrauma 38, 111–121. doi: 10.1089/neu.2019.6574, PMID: 32515269 PMC7757530

[ref18] Bartolomei F. Bosma I. Klein M. Baayen J. C. Reijneveld J. C. Postma T. J.. (2006). Disturbed functional connectivity in brain tumour patients: evaluation by graph analysis of synchronization matrices. Clin. Neurophysiol. 117, 2039–2049. doi: 10.1016/j.clinph.2006.05.018, PMID: 16859985

[ref19] Barzó P. Marmarou A. Fatouros P. Hayasaki K. Corwin F. (1997). Contribution of vasogenic and cellular edema to traumatic brain swelling measured by diffusion-weighted imaging. J. Neurosurg. 87, 900–907. doi: 10.3171/jns.1997.87.6.0900 9384402

[ref20] Bells S. Lefebvre J. Longoni G. Narayanan S. Arnold D. L. Yeh E. A.. (2019). White matter plasticity and maturation in human cognition. Glia 67, 2020–2037. doi: 10.1002/glia.23661 31233643

[ref21] Bendlin B. B. Ries M. L. Lazar M. Alexander A. L. Dempsey R. J. Rowley H. A.. (2008). Longitudinal changes in patients with traumatic brain injury assessed with diffusion-tensor and volumetric imaging. NeuroImage 42, 503–514. doi: 10.1016/j.neuroimage.2008.04.254, PMID: 18556217 PMC2613482

[ref22] Bethlehem R. A. I. Seidlitz J. White S. R. Vogel J. W. Anderson K. M. Adamson C.. (2022). Brain charts for the human lifespan. Nature 604, 525–533. doi: 10.1038/s41586-022-04554-y, PMID: 35388223 PMC9021021

[ref23] Bhardwaj R. D. Mahmoodabadi S. Z. Otsubo H. Snead O. C. Rutka J. T. Widjaja E. (2010). Diffusion tensor tractography detection of functional pathway for the spread of epileptiform activity between temporal lobe and Rolandic region. Childs Nerv. Syst. 26, 185–190. doi: 10.1007/s00381-009-1017-1, PMID: 19915854

[ref24] Binding L. P. Dasgupta D. Giampiccolo D. Duncan J. S. Vos S. B. (2022). Structure and function of language networks in temporal lobe epilepsy. Epilepsia 63, 1025–1040. doi: 10.1111/epi.17204, PMID: 35184291 PMC9773900

[ref25] Blanton R. E. Levitt J. G. Peterson J. R. Fadale D. Sporty M. L. Lee M.. (2004). Gender differences in the left inferior frontal gyrus in normal children. Neuroimage 22, 626–636. doi: 10.1016/j.neuroimage.2004.01.010, PMID: 15193591

[ref26] Bonilha L. Jensen J. H. Baker N. Breedlove J. Nesland T. Lin J. J.. (2015). The brain connectome as a personalized biomarker of seizure outcomes after temporal lobectomy. Neurology 84, 1846–1853. doi: 10.1212/WNL.0000000000001548, PMID: 25854868 PMC4433467

[ref27] Bourdillon P. Cucherat M. Isnard J. Ostrowsky-Coste K. Catenoix H. Guénot M.. (2018). Stereo-electroencephalography-guided radiofrequency thermocoagulation in patients with focal epilepsy: a systematic review and meta-analysis. Epilepsia 59, 2296–2304. doi: 10.1111/epi.14584, PMID: 30345535

[ref28] Bourdillon P. Ferrand-Sorbet S. Apra C. Chipaux M. Raffo E. Rosenberg S.. (2021). Surgical treatment of hypothalamic hamartomas. Neurosurg. Rev. 44, 753–762. doi: 10.1007/s10143-020-01298-z, PMID: 32318922

[ref29] Bourdillon P. Rheims S. Catenoix H. Montavont A. Ostrowsky-Coste K. Isnard J.. (2019). Malformations of cortical development: new surgical advances. Rev. Neurol. (Paris) 175, 183–188. doi: 10.1016/j.neurol.2019.01.392, PMID: 30819503

[ref30] Braakman H. M. H. Vaessen M. J. Jansen J. F. A. Debeij-van Hall M. H. J. A. de Louw A. Hofman P. A. M.. (2014). Pediatric frontal lobe epilepsy: White matter abnormalities and cognitive impairment. Acta Neurol. Scand. 129, 252–262. doi: 10.1111/ane.12183, PMID: 24112290

[ref31] Brauer J. Anwander A. Friederici A. D. (2011). Neuroanatomical prerequisites for language functions in the maturing brain. Cereb. Cortex 21, 459–466. doi: 10.1093/cercor/bhq108, PMID: 20566580

[ref32] Brauer J. Anwander A. Perani D. Friederici A. D. (2013). Dorsal and ventral pathways in language development. Brain Lang. 127, 289–295. doi: 10.1016/j.bandl.2013.03.001, PMID: 23643035

[ref33] Bressler S. L. Tognoli E. (2006). Operational principles of neurocognitive networks. Int J Psychophysiol [Internet]. 60, 139–148. doi: 10.1016/j.ijpsycho.2005.12.008 16490271

[ref34] Brody B. A. Kinney C. Kloman A. S. Gilles F. H. (1987). Sequence of central nervous system myelination in human infancy. I. An autopsy study of myelination. J. Neuropathol. Exp. Neurol. 46, 283–301. doi: 10.1097/00005072-198705000-00005 3559630

[ref35] Brouwer R. M. Mandl R. C. W. Schnack H. G. ILC S. Baal G. C. Peper J. S.. (2012). White matter development in early puberty: a longitudinal volumetric and diffusion tensor imaging twin study. PLoS One 7:e32316. doi: 10.1371/journal.pone.0032316, PMID: 22514599 PMC3326005

[ref36] Buyanova I. S. Arsalidou M. (2021). Cerebral White matter myelination and relations to age, gender, and cognition: a selective review. Front. Hum. Neurosci. 15:662031. doi: 10.3389/fnhum.2021.662031 34295229 PMC8290169

[ref37] Caeyenberghs K. Leemans A. De Decker C. Heitger M. Drijkoningen D. Vander Linden C.. (2012). Brain connectivity and postural control in young traumatic brain injury patients: a diffusion MRI based network analysis. Neuroimage Clin. 1, 106–115. doi: 10.1016/j.nicl.2012.09.011, PMID: 24179743 PMC3757722

[ref38] Cameron N. Bogin B. (2023). Human growth and development. J. Hum. Growth Dev., 1–582. doi: 10.1016/C2019-0-04297-5

[ref39] Cao M. Huang H. Peng Y. Dong Q. He Y. (2016). Toward developmental Connectomics of the human brain. Front. Neuroanat. 10:25. doi: 10.3389/fnana.2016.00025 27064378 PMC4814555

[ref40] Cargnelutti E. Ius T. Skrap M. Tomasino B. (2020). What do we know about pre- and postoperative plasticity in patients with glioma? A review of neuroimaging and intraoperative mapping studies. Neuroimage Clin. 28:102435. doi: 10.1016/j.nicl.2020.102435 32980599 PMC7522801

[ref41] Castellanos F. X. Cortese S. Proal E. (2014). Connectivity. Curr Top Behav Neurosci [Internet]. 16, 49–77. doi: 10.1007/7854_2013_244, PMID: 23943564

[ref42] Celtikci E. Celtikci P. Fernandes-Cabral D. T. Ucar M. Fernandez-Miranda J. C. Borcek A. O. (2017). High-definition Fiber Tractography in evaluation and surgical planning of Thalamopeduncular Pilocytic Astrocytomas in pediatric population: case series and review of literature. World Neurosurg. 98, 463–469. doi: 10.1016/j.wneu.2016.11.061, PMID: 27888085

[ref43] Chapman S. B. Max J. E. Gamino J. F. McGlothlin J. H. Cliff S. N. (2003). Discourse plasticity in children after stroke: age at injury and lesion effects. Pediatr. Neurol. 29, 34–41. doi: 10.1016/S0887-8994(03)00012-2, PMID: 13679119

[ref44] Chau Loo Kung G. Knowles J. K. Batra A. Ni L. Rosenberg J. McNab J. A. (2023). Quantitative MRI reveals widespread, network-specific myelination change during generalized epilepsy progression. Neuroimage 280:120312. doi: 10.1016/j.neuroimage.2023.120312 37574120 PMC11095339

[ref45] Chavez M. Valencia M. Navarro V. Latora V. Martinerie J. (2010). Functional modularity of background activities in normal and epileptic brain networks. Phys. Rev. Lett. 104:118701. doi: 10.1103/PhysRevLett.104.118701, PMID: 20366507

[ref46] Chen Z. Zhang H. Yushkevich P. A. Liu M. Beaulieu C. (2016). Maturation along white matter tracts in human brain using a diffusion tensor surface model tract-specific analysis. Front. Neuroanat. 10:9. doi: 10.3389/fnana.2016.00009 26909027 PMC4754466

[ref47] Cherian S. Whitelaw A. Thoresen M. Love S. (2004). The pathogenesis of neonatal post-hemorrhagic hydrocephalus. Brain Pathol. 14, 305–311. doi: 10.1111/j.1750-3639.2004.tb00069.x, PMID: 15446586 PMC8095844

[ref48] Cho J. Rahimpour S. Cutler A. Goodwin C. R. Lad S. P. Codd P. (2020). Enhancing reality: A systematic review of augmented reality in neuronavigation and education. World Neurosurg. 139, 186–195. doi: 10.1016/j.wneu.2020.04.043 32311561

[ref49] Ciumas C. Saignavongs M. Ilski F. Herbillon V. Laurent A. Lothe A.. (2014). White matter development in children with benign childhood epilepsy with centro-temporal spikes. Brain 137, 1095–1106. doi: 10.1093/brain/awu039, PMID: 24598359

[ref50] Collin G. van den Heuvel M. P. (2013). The ontogeny of the human connectome: development and dynamic changes of brain connectivity across the life span. Neuroscientist 19, 616–628. doi: 10.1177/1073858413503712, PMID: 24047610

[ref51] Concha L. Livy D. J. Beaulieu C. Wheatley B. M. Gross D. W. (2010). In vivo diffusion tensor imaging and histopathology of the fimbria-fornix in temporal lobe epilepsy. J. Neurosci. 30, 996–1002. doi: 10.1523/JNEUROSCI.1619-09.2010, PMID: 20089908 PMC6633109

[ref52] Coppola A. Tramontano V. Basaldella F. Arcaro C. Squintani G. Sala F. (2016). Intra-operative neurophysiological mapping and monitoring during brain tumour surgery in children: An update. Childs Nerv. Syst. 32, 1849–1859. doi: 10.1007/s00381-016-3180-5 27659828

[ref53] Craig B. T. Hilderley A. Kinney-Lang E. Long X. Carlson H. L. Kirton A. (2020). Developmental neuroplasticity of the white matter connectome in children with perinatal stroke. Neurol. Int. 95, E2476–E2486. doi: 10.1212/WNL.0000000000010669 PMC7682831 32887781

[ref54] Craig B. T. Kinney-Lang E. Hilderley A. J. Carlson H. L. Kirton A. (2022). Structural connectivity of the sensorimotor network within the non-lesioned hemisphere of children with perinatal stroke. Sci. Rep. 12:3866. doi: 10.1038/s41598-022-13000-y, PMID: 35264665 PMC8907195

[ref55] Crossley N. A. Fox P. T. Bullmore E. T. (2016). Meta-connectomics: human brain network and connectivity meta-analyses. Psychol Med. 46, 897–907. doi: 10.1017/S0033291715002895, PMID: 26809184 PMC8683119

[ref56] Dai X. Hadjipantelis P. Wang J. L. Deoni S. C. L. Müller H. G. (2019). Longitudinal associations between white matter maturation and cognitive development across early childhood. Hum. Brain Mapp. 40, 4130–4145. doi: 10.1002/hbm.24690, PMID: 31187920 PMC6771612

[ref57] Dallas J. Englot D. J. Naftel R. P. (2020). Neurosurgical approaches to pediatric epilepsy: indications, techniques, and outcomes of common surgical procedures. Seizure 77, 76–85. doi: 10.1016/j.seizure.2018.11.007, PMID: 30473268 PMC6522335

[ref58] David O. Maess B. Eckstein K. Friederici A. D. (2011). Dynamic causal modeling of subcortical connectivity of language. J. Neurosci. 31, 2712–2717. doi: 10.1523/JNEUROSCI.3433-10.2011, PMID: 21325540 PMC3384564

[ref59] Dayan M. Munoz M. Jentschke S. Chadwick M. J. Cooper J. M. Riney K.. (2015). Optic radiation structure and anatomy in the normally developing brain determined using diffusion MRI and tractography. Brain Struct. Funct. 220, 291–306. doi: 10.1007/s00429-013-0655-y, PMID: 24170375 PMC4286633

[ref60] De Benedictis A. Duffau H. (2011). Brain hodotopy: from esoteric concept to practical surgical applications. Neurosurgery 68, 1709–1723. doi: 10.1227/NEU.0b013e3182124690, PMID: 21346655

[ref61] De Benedictis A. Nocerino E. Menna F. Remondino F. Barbareschi M. Rozzanigo U.. (2018). Photogrammetry of the human brain: a novel method for three-dimensional quantitative exploration of the structural connectivity in neurosurgery and neurosciences. World Neurosurg. 115, e279–e291. doi: 10.1016/j.wneu.2018.04.036, PMID: 29660551

[ref62] De Benedictis A. Rossi-Espagnet M. C. de Palma L. Carai A. Marras C. E. (2022). Networking of the human cerebellum: From Anatomo-functional development to neurosurgical implications. Front. Neurol. 13:806298. doi: 10.3389/fneur.2022.806298 35185765 PMC8854219

[ref63] de Carvalho F. V. Yasuda C. L. Tedeschi G. G. Betting L. E. Cendes F. (2012). White matter abnormalities in patients with focal cortical dysplasia revealed by diffusion tensor imaging analysis in a voxelwise approach. Front. Neurol. 3:121. doi: 10.3389/fneur.2012.00121 22855684 PMC3405461

[ref64] de Faria O. Gonsalvez D. G. Nicholson M. Xiao J. (2019). Activity-dependent central nervous system myelination throughout life. J. Neurochem. 148, 447–461. doi: 10.1111/jnc.14592 30225984 PMC6587454

[ref65] de Oliveira R. S. Deriggi D. J. P. Furlanetti L. L. Santos M. V. Valera E. T. Brassesco M. S.. (2015). The impact of surgical resection of giant supratentorial brain tumor in pediatric patients: safety and neurological outcome evaluated in 23 consecutive cases. Childs Nerv. Syst. 31, 67–75. doi: 10.1007/s00381-014-2583-4, PMID: 25374270

[ref66] de Palma L. de Benedictis A. Specchio N. Marras C. E. (2020). Epileptogenic network formation. Neurosurg. Clin. N Am. 31, 335–344. doi: 10.1016/j.nec.2020.03.012 32475484

[ref67] Dean D. C. O’Muircheartaigh J. Dirks H. Waskiewicz N. Walker L. Doernberg E.. (2015). Characterizing longitudinal white matter development during early childhood. Brain Struct. Funct. 220, 1921–1933. doi: 10.1007/s00429-014-0763-3, PMID: 24710623 PMC4481335

[ref68] Debenedictis A. Marras C. E. Petit L. Sarubbo S. (2021). The inferior fronto-occipital fascicle: a century of controversies from anatomy theaters to operative neurosurgery. J. Neurosurg. Sci. 65, 605–615. doi: 10.23736/S0390-5616.21.05360-1, PMID: 33940782

[ref69] del Bigio M. R. (2010). Neuropathology and structural changes in hydrocephalus. Dev. Disabil. Res. Rev. 16, 16–22. doi: 10.1002/ddrr.94, PMID: 20419767

[ref70] Delion M. Terminassian A. Lehousse T. Aubin G. Malka J. N’Guyen S.. (2015). Specificities of awake craniotomy and brain mapping in children for resection of Supratentorial tumors in the language area. World Neurosurg. 84, 1645–1652. doi: 10.1016/j.wneu.2015.06.073, PMID: 26164190

[ref71] Dennis E. L. Jahanshad N. McMahon K. L. de Zubicaray G. I. Martin N. G. Hickie I. B.. (2013). Development of brain structural connectivity between ages 12 and 30: a 4-Tesla diffusion imaging study in 439 adolescents and adults. Neuroimage 64, 671–684. doi: 10.1016/j.neuroimage.2012.09.004 22982357 PMC3603574

[ref72] Dennis E. L. Jin Y. Villalon-Reina J. E. Zhan L. Kernan C. L. Babikian T.. (2015). White matter disruption in moderate/severe pediatric traumatic brain injury: advanced tract-based analyses. Neuroimage Clin. 7, 493–505. doi: 10.1016/j.nicl.2015.02.002, PMID: 25737958 PMC4338205

[ref73] Dennis E. L. Thompson P. M. (2013). Mapping connectivity in the developing brain. Int. J. Dev. Neurosci. 31, 525–542. doi: 10.1016/j.ijdevneu.2013.05.007, PMID: 23722009 PMC3800504

[ref74] Dennis E. L. Thompson P. M. (2014). Reprint of: mapping connectivity in the developing brain. Int. J. Dev. Neurosci. 32, 41–57. doi: 10.1016/j.ijdevneu.2013.11.005 24295552 PMC4442620

[ref75] Deoni S. C. L. Dean D. C. O’Muircheartaigh J. Dirks H. Jerskey B. A. (2012). Investigating white matter development in infancy and early childhood using myelin water faction and relaxation time mapping. Neuroimage 63, 1038–1053. doi: 10.1016/j.neuroimage.2012.07.037, PMID: 22884937 PMC3711836

[ref76] Deoni S. C. L. O’Muircheartaigh J. Elison J. T. Walker L. Doernberg E. Waskiewicz N.. (2016). White matter maturation profiles through early childhood predict general cognitive ability. Brain Struct. Funct. 221, 1189–1203. doi: 10.1007/s00429-014-0947-x, PMID: 25432771 PMC4771819

[ref77] Deshpande R. Chang L. Oishi K. (2015). Construction and application of human neonatal DTI atlases. Front. Neuroanat. 9:138. doi: 10.3389/fnana.2015.00138 26578899 PMC4620146

[ref78] Dinomais M. Hertz-Pannier L. Groeschel S. Chabrier S. Delion M. Husson B.. (2015). Long term motor function after neonatal stroke: lesion localization above all. Hum. Brain Mapp. 36, 4793–4807. doi: 10.1002/hbm.22950, PMID: 26512551 PMC6869692

[ref79] Dosenbach N. U. F. Fair D. A. Miezin F. M. Cohen A. L. Wenger K. K. Dosenbach R. A. T.. (2007). Distinct brain networks for adaptive and stable task control in humans. Proc. Natl. Acad. Sci. U. S. A. 104, 11073–11078. doi: 10.1073/pnas.0704320104, PMID: 17576922 PMC1904171

[ref80] Dubois J. Dehaene-Lambertz G. Kulikova S. Poupon C. Hüppi P. S. Hertz-Pannier L. (2014). The early development of brain white matter: a review of imaging studies in fetuses, newborns and infants. Neuroscience 276, 48–71. doi: 10.1016/j.neuroscience.2013.12.044 24378955

[ref81] Dubois J. Dehaene-Lambertz G. Soarès C. Cointepas Y. le Bihan D. Hertz-Pannier L. (2008). Microstructural correlates of infant functional development: example of the visual pathways. J. Neurosci. 28, 1943–1948. doi: 10.1523/JNEUROSCI.5145-07.2008, PMID: 18287510 PMC6671431

[ref82] Dubois J. Poupon C. Thirion B. Simonnet H. Kulikova S. Leroy F.. (2016). Exploring the early organization and maturation of linguistic pathways in the human infant brain. Cereb. Cortex 26, 2283–2298. doi: 10.1093/cercor/bhv082, PMID: 25924951

[ref83] Dubowitz L. M. S. Dubowitz V. Morante A. Verghote M. (1980). Visual function in the preterm and Fullterm newborn infant. Dev. Med. Child Neurol. 22, 465–475. doi: 10.1111/j.1469-8749.1980.tb04351.x, PMID: 7409338

[ref84] Duffau H. (2018). The error of Broca: from the traditional localizationist concept to a connectomal anatomy of human brain. J. Chem. Neuroanat. 89, 73–81. doi: 10.1016/j.jchemneu.2017.04.003, PMID: 28416459

[ref85] Duffau H. (2021a). The death of localizationism: The concepts of functional connectome and neuroplasticity deciphered by awake mapping, and their implications for best care of brain-damaged patients. Rev. Neurol. (Paris) 177, 1093–1103. doi: 10.1016/j.neurol.2021.07.016, PMID: 34563375

[ref86] Duffau H. (2021b). New philosophy, clinical pearls, and methods for intraoperative cognition mapping and monitoring “à la carte” in brain tumor patients. Neurosurgery 88, 919–930. doi: 10.1093/neuros/nyaa363, PMID: 33463689

[ref87] Duffau H. (2022). White matter tracts and diffuse lower-grade gliomas: The pivotal role of myelin plasticity in the tumor pathogenesis, infiltration patterns, functional consequences and therapeutic management. Front. Oncol. 12:855587. doi: 10.3389/fonc.2022.855587 35311104 PMC8924360

[ref88] Duffau H. Moritz-Gasser S. Mandonnet E. (2014). A re-examination of neural basis of language processing: proposal of a dynamic hodotopical model from data provided by brain stimulation mapping during picture naming. Brain Lang. 131, 1–10. doi: 10.1016/j.bandl.2013.05.011, PMID: 23866901

[ref89] Dziedzic T. A. Balasa A. Jeżewski M. P. Michałowski Ł. Marchel A. (2021). White matter dissection with the Klingler technique: a literature review. Brain Struct. Funct. 226, 13–47. doi: 10.1007/s00429-020-02157-9, PMID: 33165658 PMC7817571

[ref90] Einspieler C. Marschik P. B. Prechtl H. F. R. (2008). Human motor behavior: prenatal origin and early postnatal development. J. Psychol. 216, 147–153. doi: 10.1027/0044-3409.216.3.147

[ref91] Eluvathingal T. J. Hasan K. M. Kramer L. Fletcher J. M. Ewing-Cobbs L. (2007). Quantitative diffusion tensor tractography of association and projection fibers in normally developing children and adolescents. Cereb. Cortex 17, 2760–2768. doi: 10.1093/cercor/bhm003, PMID: 17307759 PMC2084482

[ref92] Engel J. Thompson P. M. Stern J. M. Staba R. J. Bragin A. Mody I. (2013). Connectomics and epilepsy. Curr. Opin. Neurol. 26, 186–194. doi: 10.1097/WCO.0b013e32835ee5b8, PMID: 23406911 PMC4064674

[ref93] Eriksson S. H. Rugg-Gunn F. J. Symms M. R. Barker G. J. Duncan J. S. (2001). Diffusion tensor imaging in patients with epilepsy and malformations of cortical development. Brain 124, 617–626. doi: 10.1093/brain/124.3.617, PMID: 11222460

[ref94] Evstigneev V. V. Kistsen V. V. Bulaev I. V. Sakovich R. A. (2013). The effect of structural white matter abnormalities on the clinical course of epilepsy. Adv. Clin. Exp. Med. 22, 529–537. 23986213

[ref95] Eyre J. A. Miller S. Clowry G. J. Conway E. A. Watts C. (2000). Functional corticospinal projections are established prenatally in the human foetus permitting involvement in the development of spinal motor centres. Brain 123, 51–64. doi: 10.1093/brain/123.1.51, PMID: 10611120

[ref96] Eyre J. A. Taylor J. P. Villagra F. Smith M. Miller S. (2001). Evidence of activity-dependent withdrawal of corticospinal projections during human development. Neurol. Int. 57, 1543–1554. doi: 10.1212/WNL.57.9.1543, PMID: 11706088

[ref97] Fair D. A. Cohen A. L. Power J. D. Dosenbach N. U. F. Church J. A. Miezin F. M.. (2009). Functional brain networks develop from a “local to distributed” organization. PLoS Comput. Biol. 5:e1000381. doi: 10.1371/journal.pcbi.1000381, PMID: 19412534 PMC2671306

[ref98] Feeney D. M. Baron J. C. (1986). Diaschisis. Stroke 17, 817–830. doi: 10.1161/01.STR.17.5.817, PMID: 3532434

[ref99] Feng K. Rowell A. C. Andres A. Bellando B. J. Lou X. Glasier C. M.. (2019). Diffusion tensor MRI of White matter of healthy full-term newborns: relationship to neurodevelopmental outcomes. Radiology 292, 179–187. doi: 10.1148/radiol.2019182564, PMID: 31161971 PMC6614910

[ref100] Ferracci F. X. Duffau H. (2018). Improving surgical outcome for gliomas with intraoperative mapping. Expert. Rev. Neurother. 18, 333–341. doi: 10.1080/14737175.2018.1451329 29521555

[ref101] Fields R. D. (2015). A new mechanism of nervous system plasticity: Activity-dependent myelination. Nat. Rev. Neurosci. 16, 756–767. doi: 10.1038/nrn4023 26585800 PMC6310485

[ref102] Flechsig P. (1920). Anatomie des Menschlichen Gehirns und Rückenmarks auf Myelogenetischer Grundlage. Available at: https://onlinebooks.library.upenn.edu/webbin/book/lookupid?key=olbp68390

[ref103] Flores-Justa A. Baldoncini M. Pérez Cruz J. C. Sánchez Gonzalez F. Martínez O. A. González-López P.. (2019). White matter topographic anatomy applied to temporal lobe surgery. World Neurosurg. 132, e670–e679. doi: 10.1016/j.wneu.2019.08.050, PMID: 31442654

[ref104] Foley R. Boop F. (2017). Tractography guides the approach for resection of thalamopeduncular tumors. Acta Neurochir. 159, 1597–1601. doi: 10.1007/s00701-017-3257-2, PMID: 28674731

[ref105] Foster M. T. Harishchandra L. S. Mallucci C. (2018). Pediatric central nervous system tumors: State-of-the-art and debated aspects 6, Front. Pediatr., 309, 10.3389/fped.2018.00309. 10.3389/fped.2018.00309 PMC6223202 30443540

[ref106] François C. Ripollés P. Ferreri L. Muchart J. Sierpowska J. Fons C.. (2019). Right structural and functional reorganization in four-year-old children with perinatal arterial ischemic stroke predict language production. eNeuro 6. doi: 10.1523/ENEURO.0447-18.2019, PMID: 31383726 PMC6749144

[ref107] Friederici A. D. (2012). Language development and the ontogeny of the dorsal pathway. Front. Evol. Neurosci. 4:3. doi: 10.3389/fnevo.2012.00003 22347185 PMC3272640

[ref108] Friederici A. D. Brauer J. Lohmann G. (2011). Maturation of the language network: from inter- to intrahemispheric connectivities. PLoS One 6:e20726. doi: 10.1371/journal.pone.0020726, PMID: 21695183 PMC3113799

[ref109] Friedrichs-Maeder C. L. Griffa A. Schneider J. Hüppi P. S. Truttmann A. Hagmann P. (2017). Exploring the role of white matter connectivity in cortex maturation. PLoS One 12:e0177466. doi: 10.1371/journal.pone.0177466, PMID: 28545040 PMC5435226

[ref110] Genc S. Smith R. E. Malpas C. B. Anderson V. Nicholson J. M. Efron D.. (2018). Development of white matter fibre density and morphology over childhood: a longitudinal fixel-based analysis. NeuroImage 183, 666–676. doi: 10.1016/j.neuroimage.2018.08.043, PMID: 30142448

[ref111] Geng X. Li G. Lu Z. Gao W. Wang L. Shen D.. (2017). Structural and maturational covariance in early childhood brain development. Cereb. Cortex 27, 1795–1807. doi: 10.1093/cercor/bhw022, PMID: 26874184 PMC6059236

[ref112] Geva R. Gardner J. M. Karmel B. Z. (1999). Feeding-based arousal effects on visual recognition memory in early infancy. Dev. Psychol. 35, 640–650. doi: 10.1037/0012-1649.35.3.640, PMID: 10380856

[ref113] Ghazwani Y. Patay Z. Sadighi Z. S. Sparrow J. Upadhyaya S. Boop F.. (2021). Handedness switching as a presenting sign for pediatric low-grade gliomas: An insight into brain plasticity from a short case series. J. Pediatr. Rehabil. Med. 14, 31–36. doi: 10.3233/PRM-190637, PMID: 33386828

[ref114] Giampiccolo D. Binding L. P. Caciagli L. Rodionov R. Foulon C. de Tisi J.. (2023). Thalamostriatal disconnection underpins long-term seizure freedom in frontal lobe epilepsy surgery. Brain 139, 16–17. doi: 10.1093/brain/awad085 PMC10232243 37062539

[ref115] Giampiccolo D. Parmigiani S. Basaldella F. Russo S. Pigorini A. Rosanova M.. (2021). Recording cortico-cortical evoked potentials of the human arcuate fasciculus under general anaesthesia. Clin. Neurophysiol. 132, 1966–1973. doi: 10.1016/j.clinph.2021.03.044, PMID: 34119407

[ref116] Girault J. B. Cornea E. Goldman B. D. Knickmeyer R. C. Styner M. Gilmore J. H. (2019). White matter microstructural development and cognitive ability in the first 2 years of life. Hum. Brain Mapp. 40, 1195–1210. doi: 10.1002/hbm.24439, PMID: 30353962 PMC6852619

[ref117] Glenn G. R. Jensen J. H. Helpern J. A. Spampinato M. V. Kuzniecky R. Keller S. S.. (2016). Epilepsy-related cytoarchitectonic abnormalities along white matter pathways. J. Neurol. Neurosurg. Psychiatry 87, 930–936. doi: 10.1136/jnnp-2015-312980, PMID: 27076491

[ref118] Goddings A. L. Roalf D. Lebel C. Tamnes C. K. (2021). Development of white matter microstructure and executive functions during childhood and adolescence: A review of diffusion MRI studies. Dev. Cogn. Neurosci. 51:101008. doi: 10.1016/j.dcn.2021.101008 34492631 PMC8424510

[ref119] Goldsberry G. Mitra D. MacDonald D. Patay Z. (2011). Accelerated myelination with motor system involvement in a neonate with immediate postnatal onset of seizures and hemimegalencephaly. Epilepsy Behav. 22, 391–394. doi: 10.1016/j.yebeh.2011.06.025, PMID: 21802995

[ref120] Goradia D. Chugani H. T. Govindan R. M. Behen M. Juhász C. Sood S. (2011). Reorganization of the right arcuate fasciculus following left arcuate fasciculus resection in children with intractable epilepsy. J. Child Neurol. 26, 1246–1251. doi: 10.1177/0883073811402689, PMID: 21551371

[ref121] Govaert P. Triulzi F. Dudink J. (2020). The developing brain by trimester. Handb. Clin. Neurol. 171, 245–289. doi: 10.1016/B978-0-444-64239-4.00014-X 32736754

[ref122] Govindan R. M. Chugani H. T. Makki M. I. Behen M. E. Dornbush J. Sood S. (2008). Diffusion tensor imaging of brain plasticity after occipital lobectomy. Pediatr. Neurol. 38, 27–33. doi: 10.1016/j.pediatrneurol.2007.08.004, PMID: 18054689

[ref123] Greuter L. Guzman R. Soleman J. (2021). Pediatric and adult low-grade gliomas: Where do the differences lie? Children (Basel) 8:1075. doi: 10.3390/children8111075 34828788 PMC8624473

[ref124] Gross D. W. Bastos A. Beaulieu C. (2005). Diffusion tensor imaging abnormalities in focal cortical dysplasia. Can. J. Neurol. Sci. 32, 477–482. doi: 10.1017/S0317167100004479, PMID: 16408578

[ref125] Guan J. Karsy M. Ducis K. Bollo R. J. (2016). Surgical strategies for pediatric epilepsy. Transl. Pediatr. 5, 55–66. doi: 10.21037/tp.2016.03.02, PMID: 27186522 PMC4855198

[ref126] Guillery R. W. (2005). Anatomical pathways that link perception and action. Prog. Brain Res., 235, 149–256. doi: 10.1016/S0079-6123(05)49017-2 16226588

[ref127] Günbey H. P. Ercan K. Findikoǧlu A. S. Bilir E. Karaoglanoglu M. Komurcu F.. (2011). Secondary corpus callosum abnormalities associated with antiepileptic drugs in temporal lobe epilepsy. A diffusion tensor imaging study. Neuroradiol. J. 24, 316–323. doi: 10.1177/197140091102400223, PMID: 24059625

[ref128] Habes M. Sotiras A. Erus G. Toledo J. B. Janowitz D. Wolk D. A.. (2022). White matter lesions. Neurol. Int. 91, e964–e975. Available at: https://www.ncbi.nlm.nih.gov/books/NBK562167/10.1212/WNL.0000000000006116 PMC6139818 30076276

[ref129] Hagmann P. Sporns O. Madan N. Cammoun L. Pienaar R. Wedeen V. J.. (2010). White matter maturation reshapes structural connectivity in the late developing human brain. Proc. Natl. Acad. Sci. U.S.A. 107, 19067–19072. doi: 10.1073/pnas.1009073107, PMID: 20956328 PMC2973853

[ref130] Harel H. Gordon I. Geva R. Feldman R. (2011). Gaze behaviors of preterm and full-term infants in nonsocial and social contexts of increasing dynamics: visual recognition, attention regulation, and gaze synchrony. Infancy 16, 69–90. doi: 10.1111/j.1532-7078.2010.00037.x, PMID: 32693482

[ref131] He J. Li Z. Yu Y. Lu Z. Li Z. Gong J. (2020). Cognitive function assessment and comparison on lateral ventricular tumors resection by the frontal transcortical approach and anterior transcallosal approach respectively in children. Neurosurg. Rev. 43, 619–632. doi: 10.1007/s10143-019-01088-2, PMID: 30815764

[ref132] Herbet G. (2021). Should complex cognitive functions be mapped with direct electrostimulation in wide-awake surgery? A network perspective. Front. Neurol. 12:635439. doi: 10.3389/fneur.2021.635439 33912124 PMC8072013

[ref133] Herbet G. Duffau H. (2020). Revisiting the functional anatomy of the human brain: toward a Meta-networking theory of cerebral functions. Physiol. Rev. 100, 1181–1228. doi: 10.1152/physrev.00033.2019, PMID: 32078778

[ref134] Hermoye L. Saint-Martin C. Cosnard G. Lee S. K. Kim J. Nassogne M. C.. (2006). Pediatric diffusion tensor imaging: Normal database and observation of the white matter maturation in early childhood. NeuroImage 29, 493–504. doi: 10.1016/j.neuroimage.2005.08.017, PMID: 16194615

[ref135] Herta J. Winter F. Pataraia E. Feucht M. Czech T. Porsche B.. (2022). Awake brain surgery for language mapping in pediatric patients: a single-center experience. J. Neurosurg. Pediatr. 29, 700–710. doi: 10.3171/2022.1.PEDS21569 35276657

[ref136] Holmes G. L. Ben-Ari Y. Zipursky A. (2001). The neurobiology and consequences of epilepsy in the developing brain. Pediatr. Res. 49, 320–325. doi: 10.1203/00006450-200103000-00004, PMID: 11228256

[ref137] Holt R. L. Provenzale J. M. Veerapandiyan A. Moon W. J. de Bellis M. D. Leonard S.. (2011). Structural connectivity of the frontal lobe in children with drug-resistant partial epilepsy. Epilepsy Behav. 21, 65–70. doi: 10.1016/j.yebeh.2011.03.016, PMID: 21497558 PMC3197740

[ref138] Honey C. J. Sporns O. (2008). Dynamical consequences of lesions in cortical networks. Hum. Brain Mapp. 29, 802–809. doi: 10.1002/hbm.20579, PMID: 18438885 PMC6870962

[ref139] Hong S. J. Bernhardt B. C. Gill R. S. Bernasconi N. Bernasconi A. (2017). The spectrum of structural and functional network alterations in malformations of cortical development. Brain 140, 2133–2143. doi: 10.1093/brain/awx145, PMID: 28899007

[ref140] Horgos B. Mecea M. Boer A. Szabo B. Buruiana A. Stamatian F.. (2020). White matter dissection of the fetal brain. Front. Neuroanat. 14:584266. doi: 10.3389/fnana.2020.584266 33071763 PMC7544931

[ref141] Huang H. Shu N. Mishra V. Jeon T. Chalak L. Wang Z. J.. (2015). Development of human brain structural networks through infancy and childhood. Cereb. Cortex 25, 1389–1404. doi: 10.1093/cercor/bht335, PMID: 24335033 PMC4397575

[ref142] Huang H. Zhang J. Wakana S. Zhang W. Ren T. Richards L. J.. (2006). White and gray matter development in human fetal, newborn and pediatric brains. Neuroimage 33, 27–38. doi: 10.1016/j.neuroimage.2006.06.009, PMID: 16905335

[ref143] Hunnius S. Geuze R. H. Zweens M. J. Bos A. F. (2008). Effects of preterm experience on the developing visual system: a longitudinal study of shifts of attention and gaze in early infancy. Dev. Neuropsychol. 33, 521–535. doi: 10.1080/87565640802101508, PMID: 18568902

[ref144] Husson B. Hertz-Pannier L. Renaud C. Allard D. Presles E. Landrieu P.. (2010). Motor outcomes after neonatal arterial ischemic stroke related to early MRI data in a prospective study. Pediatrics 126, 912–918. doi: 10.1542/peds.2009-3611, PMID: 20855393

[ref145] Hutchinson E. Pulsipher D. Dabbs K. Gutierrez A. M. Sheth R. Jones J.. (2010). Children with new-onset epilepsy exhibit diffusion abnormalities in cerebral white matter in the absence of volumetric differences. Epilepsy Res. 88, 208–214. doi: 10.1016/j.eplepsyres.2009.11.011 20044239 PMC2826144

[ref146] Huttenlocher P. R. de Courten C. Garey L. J. van der Loos H. (1982). Synaptogenesis in human visual cortex - evidence for synapse elimination during normal development. Neurosci. Lett. 33, 247–252. doi: 10.1016/0304-3940(82)90379-2, PMID: 7162689

[ref147] Iandolo G. Chourasia N. Ntolkeras G. Madsen J. R. Papadelis C. Grant E.. (2021). Changes in the functional brain network of children undergoing repeated epilepsy surgery: An eeg source connectivity study. Diagnostics 11:1234. doi: 10.3390/diagnostics11071234, PMID: 34359317 PMC8306224

[ref148] Ibrahim G. M. Morgan B. R. Smith M. L. Kerr E. Donner E. Go C. Y.. (2015). Thalamocortical connectivity is enhanced following functional hemispherotomy for intractable lateralized epilepsy. Epilepsy Behav. 51, 281–285. doi: 10.1016/j.yebeh.2015.07.039, PMID: 26318790

[ref149] Imamura H. Matsumoto R. Takaya S. Nakagawa T. Shimotake A. Kikuchi T.. (2016). Network specific change in white matter integrity in mesial temporal lobe epilepsy. Epilepsy Res. 120, 65–72. doi: 10.1016/j.eplepsyres.2015.12.003, PMID: 26735187

[ref150] Imms P. Clemente A. Cook M. D’Souza W. Wilson P. H. Jones D. K.. (2019). The structural connectome in traumatic brain injury: a meta-analysis of graph metrics. Neurosci. Biobehav. Rev. 99, 128–137. doi: 10.1016/j.neubiorev.2019.01.002, PMID: 30615935 PMC7615245

[ref151] Isaacs A. M. Shimony J. S. Morales D. M. Castaneyra-Ruiz L. Hartman A. Cook M.. (2019). Feasibility of fast brain diffusion MRI to quantify white matter injury in pediatric hydrocephalus. J. Neurosurg. Pediatr. 24, 461–468. doi: 10.3171/2019.5.PEDS18596, PMID: 31323624 PMC6982356

[ref152] Ius T. Angelini E. Thiebaut de Schotten M. Mandonnet E. Duffau H. (2011). Evidence for potentials and limitations of brain plasticity using an atlas of functional resectability of WHO grade II gliomas: towards a “minimal common brain”. Neuroimage 56, 992–1000. doi: 10.1016/j.neuroimage.2011.03.022, PMID: 21414413

[ref153] James J. S. Radhakrishnan A. Thomas B. Madhusoodanan M. Kesavadas C. Abraham M.. (2015). Diffusion tensor imaging tractography of Meyer’s loop in planning resective surgery for drug-resistant temporal lobe epilepsy. Epilepsy Res. 110, 95–104. doi: 10.1016/j.eplepsyres.2014.11.020, PMID: 25616461

[ref154] Jeong J. W. Asano E. Juhász C. Behen M. E. Chugani H. T. (2016). Postoperative axonal changes in the contralateral hemisphere in children with medically refractory epilepsy: a longitudinal diffusion tensor imaging connectome analysis. Hum. Brain Mapp. 37, 3946–3956. doi: 10.1002/hbm.23287, PMID: 27312605 PMC5053859

[ref155] Jeong J. W. Asano E. Juhász C. Chugani H. T. (2015). Localization of specific language pathways using diffusion-weighted imaging tractography for presurgical planning of children with intractable epilepsy. Epilepsia 56, 49–57. doi: 10.1111/epi.12863, PMID: 25489639 PMC4354866

[ref156] Ji G. J. Zhang Z. Xu Q. Wei W. Wang J. Wang Z.. (2015). Connectome reorganization associated with surgical outcome in temporal lobe epilepsy. Medicine 94:e1737. doi: 10.1097/MD.0000000000001737, PMID: 26448031 PMC4616737

[ref157] Jin C. Li Y. Li X. Wang M. Liu C. Gao J.. (2019). Proper timing for the evaluation of neonatal brain white matter development: a diffusion tensor imaging study. Eur. Radiol. 29, 1527–1537. doi: 10.1007/s00330-018-5665-y, PMID: 30151640

[ref158] Jirsa V. Wang H. Triebkorn P. Hashemi M. Jha J. Gonzalez-Martinez J.. (2023). Personalised virtual brain models in epilepsy. Lancet Neurol. 22, 443–454. doi: 10.1016/S1474-4422(23)00008-X 36972720

[ref159] Keller S. S. Ahrens T. Mohammadi S. Möddel G. Kugel H. Bernd Ringelstein E.. (2011). Microstructural and volumetric abnormalities of the putamen in juvenile myoclonic epilepsy. Epilepsia 52, 1715–1724. doi: 10.1111/j.1528-1167.2011.03117.x, PMID: 21635242

[ref160] Khan O. H. Enno T. L. del Bigio M. R. (2006). Brain damage in neonatal rats following kaolin induction of hydrocephalus. Exp. Neurol. 200, 311–320. doi: 10.1016/j.expneurol.2006.02.113, PMID: 16624304

[ref161] Khundrakpam B. S. Lewis J. D. Zhao L. Chouinard-Decorte F. Evans A. C. (2016). Brain connectivity in normally developing children and adolescents. Neuroimage 134, 192–203. doi: 10.1016/j.neuroimage.2016.03.062, PMID: 27054487

[ref162] Kim D. J. Davis E. P. Sandman C. A. Sporns O. O’Donnell B. F. Buss C.. (2014). Longer gestation is associated with more efficient brain networks in preadolescent children. NeuroImage 100, 619–627. doi: 10.1016/j.neuroimage.2014.06.048, PMID: 24983711 PMC4138264

[ref163] Kim D. J. Davis E. P. Sandman C. A. Sporns O. O’Donnell B. F. Buss C.. (2016). Children’s intellectual ability is associated with structural network integrity. NeuroImage 124, 550–556. doi: 10.1016/j.neuroimage.2015.09.012, PMID: 26385010 PMC4651770

[ref164] Kimiwada T. Juhász C. Makki M. Muzik O. Chugani D. C. Asano E.. (2006). Hippocampal and thalamic diffusion abnormalities in children with temporal lobe epilepsy. Epilepsia 47, 167–175. doi: 10.1111/j.1528-1167.2006.00383.x, PMID: 16417545

[ref165] Kin T. Nakatomi H. Shono N. Nomura S. Saito T. Oyama H.. (2017). Neurosurgical virtual reality simulation for brain tumor using high-definition computer graphics: A review of the literature. Neurol. Med. Chir. 57, 513–520. doi: 10.2176/nmc.ra.2016-0320 PMC5638778 28637947

[ref166] Kinney H. C. Brody B. A. Kloman A. S. Gilles F. H. (1988). Sequence of central nervous system myelination in human infancy. II. Patterns of myelination in autopsied infants. J. Neuropathol. Exp. Neurol. 47, 217–234. doi: 10.1097/00005072-198805000-00003 3367155

[ref167] Kinnunen K. M. Greenwood R. Powell J. H. Leech R. Hawkins P. C. Bonnelle V.. (2011). White matter damage and cognitive impairment after traumatic brain injury. Brain 134, 449–463. doi: 10.1093/brain/awq347, PMID: 21193486 PMC3030764

[ref168] Kirton A. Metzler M. J. Craig B. T. Hilderley A. Dunbar M. Giuffre A.. (2021). Perinatal stroke: mapping and modulating developmental plasticity. Nat. Rev. Neurol. 17, 415–432. doi: 10.1038/s41582-021-00503-x, PMID: 34127850

[ref169] Klinger J. (1935). Erleichterung der makroskopischen präparation des gehirn durch den gefrierprozess. Schweiz Arch. Neurol., 247, 36–256.

[ref170] Knowles J. K. Batra A. Xu H. Monje M. (2022). Adaptive and maladaptive myelination in health and disease. Nat. Rev. Neurol. 18, 735–746. doi: 10.1038/s41582-022-00737-3, PMID: 36376595

[ref171] Koenis M. M. G. Brouwer R. M. van den Heuvel M. P. Mandl R. C. W. van Soelen I. L. C. Kahn R. S.. (2015). Development of the brain’s structural network efficiency in early adolescence: a longitudinal DTI twin study. Hum. Brain Mapp. 36, 4938–4953. doi: 10.1002/hbm.22988, PMID: 26368846 PMC6869380

[ref172] Königs M. van Heurn L. W. E. Bakx R. Vermeulen R. J. Goslings J. C. Poll-The B. T.. (2017). The structural connectome of children with traumatic brain injury. Hum. Brain Mapp. 38, 3603–3614. doi: 10.1002/hbm.23614, PMID: 28429381 PMC6866988

[ref173] Kraus M. F. Susmaras T. Caughlin B. P. Walker C. J. Sweeney J. A. Little D. M. (2007). White matter integrity and cognition in chronic traumatic brain injury: a diffusion tensor imaging study. Brain 130, 2508–2519. doi: 10.1093/brain/awm216, PMID: 17872928

[ref174] Kreilkamp B. A. K. McKavanagh A. Alonazi B. Bryant L. Das K. Wieshmann U. C.. (2021). Altered structural connectome in non-lesional newly diagnosed focal epilepsy: relation to pharmacoresistance. Neuroimage Clin. 29:102564. doi: 10.1016/j.nicl.2021.102564 33508622 PMC7841400

[ref175] Krogsrud S. K. Fjell A. M. Tamnes C. K. Grydeland H. Mork L. Due-Tønnessen P.. (2016). Changes in white matter microstructure in the developing brain-a longitudinal diffusion tensor imaging study of children from 4 to 11years of age. NeuroImage 124, 473–486. doi: 10.1016/j.neuroimage.2015.09.017, PMID: 26375208 PMC4655940

[ref176] Kucukyuruk B. Richardson R. M. Wen H. T. Fernandez-Miranda J. C. Rhoton A. L. (2012). Microsurgical anatomy of the temporal lobe and its implications on temporal lobe epilepsy surgery. Epilepsy Res. Treat. 2012, 1–17. doi: 10.1155/2012/769825, PMID: 22957242 PMC3420566

[ref177] Kucukyuruk B. Yagmurlu K. Tanriover N. Uzan M. Rhoton A. L. (1982). Microsurgical anatomy of the white matter tracts in hemispherotomy. Neurosurgery 10, 305–324. doi: 10.1227/NEU.0000000000000288 24448186

[ref178] Kulikova S. Hertz-Pannier L. Dehaene-Lambertz G. Buzmakov A. Poupon C. Dubois J. (2015). Multi-parametric evaluation of the white matter maturation. Brain Struct. Funct. 220, 3657–3672. doi: 10.1007/s00429-014-0881-y, PMID: 25183543 PMC4575699

[ref179] Küpper H. Kudernatsch M. Pieper T. Groeschel S. Tournier J. D. Raffelt D.. (2016). Predicting hand function after hemidisconnection. Brain 139, 2456–2468. doi: 10.1093/brain/aww170, PMID: 27383529

[ref180] Lacerda L. M. Clayden J. D. Handley S. E. Winston G. P. Kaden E. Tisdall M.. (2020). Microstructural investigations of the visual pathways in pediatric epilepsy neurosurgery: insights from multi-Shell diffusion magnetic resonance imaging. Front. Neurosci. 14:269. doi: 10.3389/fnins.2020.00269 32322185 PMC7158873

[ref181] Lebel C. Beaulieu C. (2011). Longitudinal development of human brain wiring continues from childhood into adulthood. J. Neurosci. 31, 10937–10947. doi: 10.1523/JNEUROSCI.5302-10.2011 21795544 PMC6623097

[ref182] Lebel C. Deoni S. (2018). The development of brain white matter microstructure. NeuroImage 182, 207–218. doi: 10.1016/j.neuroimage.2017.12.097 29305910 PMC6030512

[ref183] Lebel C. Treit S. Beaulieu C. (2019). A review of diffusion MRI of typical white matter development from early childhood to young adulthood. NMR Biomed. 32:e3778. doi: 10.1002/nbm.3778, PMID: 28886240

[ref184] Lee M. J. Kim H. D. Lee J. S. Kim D. S. Lee S. K. (2013). Usefulness of diffusion tensor tractography in pediatric epilepsy surgery. Yonsei Med. J. 54, 21–27. doi: 10.3349/ymj.2013.54.1.21, PMID: 23225794 PMC3521255

[ref185] Lee C. Y. Tabesh A. Spampinato M. V. Helpern J. A. Jensen J. H. Bonilha L. (2014). Diffusional kurtosis imaging reveals a distinctive pattern of microstructural alternations in idiopathic generalized epilepsy. Acta Neurol. Scand. 130, 148–155. doi: 10.1111/ane.12257, PMID: 24796428 PMC4134765

[ref186] Li L. Liu J. (2013). The effect of pediatric traumatic brain injury on behavioral outcomes: a systematic review. Dev. Med. Child Neurol. 55, 37–45. doi: 10.1111/j.1469-8749.2012.04414.x, PMID: 22998525 PMC3593091

[ref187] Li Y. Wang Y. Tan Z. Chen Q. Huang W. (2018). Longitudinal brain functional and structural connectivity changes after hemispherotomy in two pediatric patients with drug-resistant epilepsy. Epilepsy Behav. Case Rep. 11, 58–66. doi: 10.1016/j.ebcr.2018.11.003 30723671 PMC6350230

[ref188] Li N. Yang Y. Glover D. P. Zhang J. Saraswati M. Robertson C.. (2014). Evidence for impaired plasticity after traumatic brain injury in the developing brain. J. Neurotrauma 31, 395–403. doi: 10.1089/neu.2013.3059 24050267 PMC3922417

[ref189] Liu M. Gross D. W. Wheatley B. M. Concha L. Beaulieu C. (2013). The acute phase of Wallerian degeneration: longitudinal diffusion tensor imaging of the fornix following temporal lobe surgery. NeuroImage 74, 128–139. doi: 10.1016/j.neuroimage.2013.01.069, PMID: 23396161

[ref190] Liu F. Scantlebury N. Tabori U. Bouffet E. Laughlin S. Strother D.. (2015). White matter compromise predicts poor intellectual outcome in survivors of pediatric low-grade glioma. Neuro. Oncol. 17, 604–613. doi: 10.1093/neuonc/nou306, PMID: 25395463 PMC4483078

[ref191] Lohkamp L. N. Beuriat P. A. Desmurget M. Cristofori I. Szathmari A. Huguet L.. (2020). Awake brain surgery in children—a single-center experience. Childs Nerv. Syst. 36, 967–974. doi: 10.1007/s00381-020-04522-9, PMID: 32055975

[ref192] Lohkamp L. N. Mottolese C. Szathmari A. Huguet L. Beuriat P. A. Christofori I.. (2019). Awake brain surgery in children—Review of the literature and state-of-the-art. Childs Nerv. Syst. 35, 2071–2077. doi: 10.1007/s00381-019-04279-w 31377911

[ref193] Loubinoux I. Brihmat N. Castel-Lacanal E. Marque P. (2017). Cerebral imaging of post-stroke plasticity and tissue repair. Rev. Neurol. (Paris) 173, 577–583. doi: 10.1016/j.neurol.2017.09.007, PMID: 28985963

[ref194] Mackay M. T. Chen J. Shapiro J. Manuela P.-W. Slavova N. Grunt S.. (2023). Association of acute infarct topography with development of cerebral palsy and neurological impairment in neonates with stroke. Neurol. Int. 101, e1509–e1520. doi: 10.1212/WNL.0000000000207705, PMID: 37591776 PMC10585702

[ref195] Marmarou A. Signoretti S. Fatouros P. P. Portella G. Aygok G. A. Bullock M. R. (2006). Predominance of cellular edema in traumatic brain swelling in patients with severe head injuries. J. Neurosurg. 104, 720–730. doi: 10.3171/jns.2006.104.5.720, PMID: 16703876

[ref196] Matsumoto R. Nair D. R. LaPresto E. Najm I. Bingaman W. Shibasaki H.. (2004). Functional connectivity in the human language system: a cortico-cortical evoked potential study. Brain 127, 2316–2330. doi: 10.1093/brain/awh246, PMID: 15269116

[ref197] McDonald C. R. Ahmadi M. E. Hagler D. J. Tecoma E. S. Iragui V. J. Gharapetian L.. (2008). Diffusion tensor imaging correlates of memory and language impairments in temporal lobe epilepsy. Neurology 71, 1869–1876. doi: 10.1212/01.wnl.0000327824.05348.3b, PMID: 18946001 PMC2676974

[ref198] McEvoy S. D. Lee A. Poliakov A. Friedman S. Shaw D. Browd S. R.. (2016). Longitudinal cerebellar diffusion tensor imaging changes in posterior fossa syndrome. Neuroimage Clin. 12, 582–590. doi: 10.1016/j.nicl.2016.09.007, PMID: 27689022 PMC5031477

[ref199] McIntosh A. R. (2000). Towards a network theory of cognition. Neural Netw. 13, 861–870. doi: 10.1016/s0893-6080(00)00059-9 11156197

[ref200] McKenzie I. A. Ohayon D. Li H. De Faria J. P. Emery B. Tohyama K.. (2014). Motor skill learning requires active central myelination. Science 346, 318–322. doi: 10.1126/science.1254960, PMID: 25324381 PMC6324726

[ref201] Meoded A. Huisman T. A. G. M. Casamassima M. G. S. Jallo G. I. Poretti A. (2017). The structural connectome in children: basic concepts, how to build it, and synopsis of challenges for the developing pediatric brain. Neuroradiology 59, 445–460. doi: 10.1007/s00234-017-1831-1, PMID: 28382501

[ref202] Minardi C. Minacapelli R. Valastro P. Vasile F. Pitino S. Pavone P.. (2019). Epilepsy in children: from diagnosis to treatment with focus on emergency. J. Clin. Med. 8:39. doi: 10.3390/jcm8010039 30609770 PMC6352402

[ref203] Mishra V. Cheng H. Gong G. He Y. Dong Q. Huang H. (2013). Differences of inter-tract correlations between neonates and children around puberty: a study based on microstructural measurements with DTI. Front. Hum. Neurosci. 7:721. doi: 10.3389/fnhum.2013.00721 24194711 PMC3810597

[ref204] Mito R. Vaughan D. N. Semmelroch M. Connelly A. Jackson G. D. (2022). Bilateral structural network abnormalities in epilepsy associated with bottom-of-sulcus dysplasia. Neurology 98, E152–E163. doi: 10.1212/WNL.0000000000013006 34675097 PMC8762587

[ref205] Moguilner S. Birba A. Fino D. Isoardi R. Huetagoyena C. Otoya R.. (2021). Structural and functional motor-network disruptions predict selective action-concept deficits: evidence from frontal lobe epilepsy. Cortex 144, 43–55. doi: 10.1016/j.cortex.2021.08.003, PMID: 34637999 PMC8585706

[ref206] Mohades S. G. van Schuerbeek P. Rosseel Y. van de Craen P. Luypaert R. Baeken C. (2015). White-matter development is different in bilingual and monolingual children: a longitudinal DTI study. PLoS One 10:e0117968. doi: 10.1371/journal.pone.0117968, PMID: 25706865 PMC4338107

[ref207] Mohammad S. A. Nashaat N. H. (2017). Age-related changes of white matter association tracts in normal children throughout adulthood: a diffusion tensor tractography study. Neuroradiology 59, 715–724. doi: 10.1007/s00234-017-1858-3, PMID: 28580531

[ref208] Moore J. K. Linthicum F. H. (2007). The human auditory system: a timeline of development. Int. J. Audiol. 46, 460–478. doi: 10.1080/14992020701383019 17828663

[ref209] Nagesh V. Tsien C. I. Chenevert T. L. Ross B. D. Lawrence T. S. Junick L.. (2008). Radiation-induced changes in normal-appearing white matter in patients with cerebral tumors: a diffusion tensor imaging study. Int. J. Radiat. Oncol. Biol. Phys. 70, 1002–1010. doi: 10.1016/j.ijrobp.2007.08.020 18313524 PMC2376211

[ref210] Nagy Z. Westerberg H. Klingberg T. (2004). Maturation of white matter is associated with the development of cognitive functions during childhood. J. Cogn. Neurosci. 16, 1227–1233. doi: 10.1162/0898929041920441 15453975

[ref211] Nevalainen P. Lauronen L. Pihko E. (2014). Development of human somatosensory cortical functions - what have we learned from magnetoencephalography: A review. Front. Hum. Neurosci. 8, 8:158. doi: 10.3389/fnhum.2014.00158 24672468 PMC3955943

[ref212] Nieberlein L. Rampp S. Gussew A. Prell J. Hartwigsen G. (2023). Reorganization and plasticity of the language network in patients with cerebral gliomas. Neuroimage Clin. 37:103326. doi: 10.1016/j.nicl.2023.103326 36736198 PMC9926312

[ref213] Northam G. B. Adler S. Eschmann K. C. J. Chong W. K. Cowan F. M. Baldeweg T. (2018). Developmental conduction aphasia after neonatal stroke. Ann. Neurol. 83, 664–675. doi: 10.1002/ana.25218, PMID: 29572915 PMC6681109

[ref214] Olopade F. E. Shokunbi M. T. Sirén A. L. (2012). The relationship between ventricular dilatation, neuropathological and neurobehavioural changes in hydrocephalic rats. Fluids Barriers CNS 9:19. doi: 10.1186/2045-8118-9-19, PMID: 22938200 PMC3464139

[ref215] Ostrom Q. T. Gittleman H. Stetson L. Virk S. M. Barnholtz-Sloan J. S. (2015). Epidemiology of gliomas. Cancer Treat Res. 163, 1–14. doi: 10.1007/978-3-319-12048-5_1 25468222

[ref216] Otte W. M. Van Eijsden P. Sander J. W. Duncan J. S. Dijkhuizen R. M. Braun K. P. J. (2012). A meta-analysis of white matter changes in temporal lobe epilepsy as studied with diffusion tensor imaging. Epilepsia 53, 659–667. doi: 10.1111/j.1528-1167.2012.03426.x, PMID: 22379949

[ref217] Ouyang M. Kang H. Detre J. A. Roberts T. P. L. Huang H. (2017). Short-range connections in the developmental connectome during typical and atypical brain maturation. Neurosci. Biobehav. Rev. 83, 109–122. doi: 10.1016/j.neubiorev.2017.10.007, PMID: 29024679 PMC5730465

[ref218] Özyurt J. Müller H. L. Warmuth-Metz M. Thiel C. M. (2017). Hypothalamic tumors impact gray and white matter volumes in fronto-limbic brain areas. Cortex 1, 98–110. doi: 10.1016/j.cortex.2017.01.017 28259055

[ref219] Pal D. Gupta R. K. Agarwal S. Yadav A. Ojha B. K. Awasthi A.. (2012). Diffusion tensor tractography indices in patients with frontal lobe injury and its correlation with neuropsychological tests. Clin. Neurol. Neurosurg. 114, 564–571. doi: 10.1016/j.clineuro.2011.12.002, PMID: 22209144

[ref220] Paldino M. J. Golriz F. Chapieski M. L. Zhang W. Chu Z. D. (2017). Brain network architecture and global intelligence in children with focal epilepsy. AJNR Am. J. Neuroradiol. 38, 349–356. doi: 10.3174/ajnr.A4975 27737853 PMC7963842

[ref221] Paldino M. J. Hedges K. Zhang W. (2014). Independent contribution of individual white matter pathways to language function in pediatric epilepsy patients. Neuroimage Clin. 6, 327–332. doi: 10.1016/j.nicl.2014.09.017, PMID: 25379446 PMC4215459

[ref222] Park H. J. Friston K. (1979). Structural and functional brain networks: from connections to cognition. Science 342. doi: 10.1126/science.1238411 24179229

[ref223] Parker C. S. Clayden J. D. Cardoso M. J. Rodionov R. Duncan J. S. Scott C.. (2018). Structural and effective connectivity in focal epilepsy. Neuroimage Clin. 17, 943–952. doi: 10.1016/j.nicl.2017.12.020, PMID: 29527498 PMC5842760

[ref224] Parks E. L. Madden D. J. (2013). Brain connectivity and visual attention. Brain Connect. 3, 317–338. doi: 10.1089/brain.2012.0139, PMID: 23597177 PMC3749701

[ref225] Paus T. (1998). Imaging the brain before, during, and after transcranial magnetic stimulation. Neuropsychologia 37, 219–224. doi: 10.1016/S0028-3932(98)00096-7 10080379

[ref226] Paus T. Collins D. L. Evans A. C. Leonard G. Pike B. Zijdenbos A. (2001). Maturation of white matter in the human brain: A review of magnetic resonance studies. Brain Res. Bull. 54, 255–266. doi: 10.1016/s0361-9230(00)00434-2 11287130

[ref227] Perani D. Saccuman M. C. Scifo P. Awander A. Spada D. Baldoli C.. (2011). Neural language networks at birth. Proc. Natl. Acad. Sci. U. S. A. 108, 16056–16061. doi: 10.1073/pnas.1102991108, PMID: 21896765 PMC3179044

[ref228] Peters B. D. Ikuta T. Derosse P. John M. Burdick K. E. Gruner P.. (2014). Age-related differences in white matter tract microstructure are associated with cognitive performance from childhood to adulthood. Biol. Psychiatry 75, 248–256. doi: 10.1016/j.biopsych.2013.05.020, PMID: 23830668 PMC4412928

[ref229] Peterson R. K. Tabori U. Bouffet E. Laughlin S. Liu F. Scantlebury N.. (2019). Predictors of neuropsychological late effects and white matter correlates in children treated for a brain tumor without radiation therapy. Pediatr. Blood Cancer 66:e27924. doi: 10.1002/pbc.27924, PMID: 31309694

[ref230] Powell H. W. R. Parker G. J. M. Alexander D. C. Symms M. R. Boulby P. A. Wheeler-Kingshott C. A. M.. (2007). Abnormalities of language networks in temporal lobe epilepsy. NeuroImage 36, 209–221. doi: 10.1016/j.neuroimage.2007.02.028, PMID: 17400477

[ref231] Prastawa M. Sadeghi N. Gilmore J. H. Lin W. Gerig G. (2010). A new framework for analyzing white matter maturation in early brain development. Proc. IEEE Int. Symp. Biomed. Imaging 2010, 97–100. doi: 10.1109/isbi.2010.5490404, PMID: 23959442 PMC3744242

[ref232] Pujar S. S. Seunarine K. K. Martinos M. M. Neville B. G. R. Scott R. C. Chin R. F. M.. (2017). Long-term white matter tract reorganization following prolonged febrile seizures. Epilepsia 58, 772–780. doi: 10.1111/epi.13724, PMID: 28332711 PMC5484997

[ref233] Pujol J. Soriano-Mas C. Ortiz H. Sebastián-Gallés N. Losilla J. M. Deus J. (2006). Myelination of language-related areas in the developing brain. Neurology 66, 339–343. doi: 10.1212/01.wnl.0000201049.66073.8d, PMID: 16476931

[ref234] Qi T. Schaadt G. Cafiero R. Brauer J. Skeide M. A. Friederici A. D. (2019). The emergence of long-range language network structural covariance and language abilities. Neuroimage 191, 36–48. doi: 10.1016/j.neuroimage.2019.02.014, PMID: 30738206

[ref235] Radhakrishnan A. James J. S. Kesavadas C. Thomas B. Bahuleyan B. Abraham M.. (2011). Utility of diffusion tensor imaging tractography in decision making for extratemporal resective epilepsy surgery. Epilepsy Res. 97, 52–63. doi: 10.1016/j.eplepsyres.2011.07.003 21835594

[ref236] Ratha V. Sampath N. Subramaniam S. Kumar V. R. R. (2021). Technical considerations in awake craniotomy with cortical and subcortical motor mapping in preadolescents: pushing the envelope. Pediatr. Neurosurg. 56, 171–178. doi: 10.1159/000513004, PMID: 33756468

[ref237] Raybaud C. (2016). Cerebral hemispheric low-grade glial tumors in children: Preoperative anatomic assessment with MRI and DTI. Childs Nerv. Syst. 32, 1799–1811. doi: 10.1007/s00381-016-3188-x 27659823

[ref238] Reddick W. E. White H. A. Glass J. O. Wheeler G. C. Thompson S. J. Gajjar A.. (2003). Developmental model relating white matter volume to neurocognitive deficits in pediatric brain tumor survivors. Cancer 97, 2512–2519. doi: 10.1002/cncr.11355, PMID: 12733151

[ref239] Rehder R. Abd-El-Barr M. Hooten K. Weinstock P. Madsen J. R. Cohen A. R. (2016). The role of simulation in neurosurgery. Childs Nerv. Syst. 32, 43–54. doi: 10.1007/s00381-015-2923-z, PMID: 26438547

[ref240] Reijneveld J. C. Ponten S. C. Berendse H. W. Stam C. J. (2007). The application of graph theoretical analysis to complex networks in the brain. Clin. Neurophysiol. 118, 2317–2331. doi: 10.1016/j.clinph.2007.08.010, PMID: 17900977

[ref241] Rezayev A. Feldman H. A. Levman J. Takahashi E. (2018). Bilateral thalamocortical abnormalities in focal cortical dysplasia. Brain Res. 1694, 38–45. doi: 10.1016/j.brainres.2018.05.005, PMID: 29738718

[ref242] Richardson M. P. (2012). Large scale brain models of epilepsy: dynamics meets connectomics. J. Neurol. Neurosurg. Psychiatry 83, 1238–1248. doi: 10.1136/jnnp-2011-301944, PMID: 22917671

[ref243] Riley J. D. Franklin D. L. Choi V. Kim R. C. Binder D. K. Cramer S. C.. (2010). Altered white matter integrity in temporal lobe epilepsy: association with cognitive and clinical profiles. Epilepsia 51, 536–545. doi: 10.1111/j.1528-1167.2009.02508.x, PMID: 20132296 PMC2929974

[ref244] Robinson K. E. Fraley C. E. Pearson M. M. Kuttesch J. F. Compas B. E. (2013). Neurocognitive late effects of pediatric brain tumors of the posterior fossa: a quantitative review. J. Int. Neuropsychol. Soc. 19, 44–53. doi: 10.1017/S1355617712000987 23095276

[ref245] Roger E. Petit L. Perrone-Bertolotti M. Job A. S. Minotti L. Kahane P.. (2018). The link between structural connectivity and neurocognition illustrated by focal epilepsy. Epileptic Disord. 20, 88–98. doi: 10.1684/epd.2018.0958, PMID: 29620009

[ref246] Rosenstock T. Picht T. Schneider H. Vajkoczy P. Thomale U. W. (2020). Pediatric navigated transcranial magnetic stimulation motor and language mapping combined with diffusion tensor imaging tractography: clinical experience. J. Neurosurg. Pediatr. 26, 583–593. doi: 10.3171/2020.4.PEDS20174, PMID: 32707554

[ref247] Rosselli M. Ardila A. Matute E. Vélez-Uribe I. (2014). Language development across the life span: a neuropsychological/neuroimaging perspective. Neurosci. J. 2014, 1–21. doi: 10.1155/2014/585237, PMID: 26317109 PMC4437268

[ref248] Roth J. Korn A. Sala F. Benvenisti H. Jubran M. Bitan-Talmor Y.. (2020). Intraoperative neurophysiology in pediatric supratentorial surgery: experience with 57 cases. Childs Nerv. Syst. 36, 315–324. doi: 10.1007/s00381-019-04356-0, PMID: 31422426

[ref249] Rueckriegel S. M. Bruhn H. Thomale U. W. Hernáiz D. P. (2015). Cerebral white matter fractional anisotropy and tract volume as measured by MR imaging are associated with impaired cognitive and motor function in pediatric posterior fossa tumor survivors. Pediatr. Blood Cancer 62, 1252–1258. doi: 10.1002/pbc.25485, PMID: 25850573

[ref250] Sarubbo S. de Benedictis A. Merler S. Mandonnet E. Balbi S. Granieri E.. (2015). Towards a functional atlas of human white matter. Hum. Brain Mapp. 36, 3117–3136. doi: 10.1002/hbm.22832, PMID: 25959791 PMC6869563

[ref251] Sarubbo S. de Benedictis A. Merler S. Mandonnet E. Barbareschi M. Dallabona M.. (2016). Structural and functional integration between dorsal and ventral language streams as revealed by blunt dissection and direct electrical stimulation. Hum. Brain Mapp. 37, 3858–3872. doi: 10.1002/hbm.23281, PMID: 27258125 PMC6867442

[ref252] Sarubbo S. Tate M. de Benedictis A. Merler S. Moritz-Gasser S. Herbet G.. (2020). Mapping critical cortical hubs and white matter pathways by direct electrical stimulation: an original functional atlas of the human brain. NeuroImage 205:116237. doi: 10.1016/j.neuroimage.2019.116237, PMID: 31626897 PMC7217287

[ref253] Sharp D. J. Scott G. Leech R. (2014). Network dysfunction after traumatic brain injury. Nat. Rev. Neurol. 10, 156–166. doi: 10.1038/nrneurol.2014.15, PMID: 24514870

[ref254] Shon Y. M. Kim Y. I. Koo B. B. Lee J. M. Kim H. J. Kim W. J.. (2010). Group-specific regional white matter abnormality revealed in diffusion tensor imaging of medial temporal lobe epilepsy without hippocampal sclerosis. Epilepsia 51, 529–535. doi: 10.1111/j.1528-1167.2009.02327.x, PMID: 19817819

[ref255] Sinha N. Dauwels J. Kaiser M. Cash S. S. Westover M. B. Wang Y.. (2017). Predicting neurosurgical outcomes in focal epilepsy patients using computational modelling. Brain 140, 319–332. doi: 10.1093/brain/aww299 28011454 PMC5278304

[ref256] Skeide M. A. Friederici A. D. (2016). The ontogeny of the cortical language network. Nat. Rev. Neurosci. 17, 323–332. doi: 10.1038/nrn.2016.23, PMID: 27040907

[ref257] Skirrow C. Cross J. H. Harrison S. Cormack F. Harkness W. Coleman R.. (2015). Temporal lobe surgery in childhood and neuroanatomical predictors of long-term declarative memory outcome. Brain 138, 80–93. doi: 10.1093/brain/awu313, PMID: 25392199 PMC4285190

[ref258] Slinger G. Sinke M. R. T. Braun K. P. J. Otte W. M. (2016). White matter abnormalities at a regional and voxel level in focal and generalized epilepsy: a systematic review and meta-analysis. Neuroimage Clin. 12, 902–909. doi: 10.1016/j.nicl.2016.10.025, PMID: 27882296 PMC5114611

[ref259] Song L. Mishra V. Ouyang M. Peng Q. Slinger M. Liu S.. (2017). Human fetal brain connectome: structural network development from middle fetal stage to birth. Front. Neurosci. 11:561. doi: 10.3389/fnins.2017.00561 29081731 PMC5645529

[ref260] Sonoda M. Silverstein B. H. Jeong J. W. Sugiura A. Nakai Y. Mitsuhashi T.. (2021). Six-dimensional dynamic tractography atlas of language connectivity in the developing brain. Brain 144, 3340–3354. doi: 10.1093/brain/awab225, PMID: 34849596 PMC8677551

[ref261] Sotardi S. Gollub R. L. Bates S. Weiss R. Murphy S. N. Grant P. E.. (2021). Voxelwise and regional brain apparent diffusion coefficient changes on MRI from birth to 6 years of age. Radiology 298, 415–424. doi: 10.1148/radiol.2020202279, PMID: 33289612 PMC7850240

[ref262] Sporns O. (2013). The human connectome: origins and challenges. NeuroImage 80, 53–61. doi: 10.1016/j.neuroimage.2013.03.023, PMID: 23528922

[ref263] Sporns O. (2015). Cerebral cartography and connectomics. Philos. Trans. R Soc. Lond. B Biol. Sci. 370:20140173. doi: 10.1098/rstb.2014.0173 25823870 PMC4387514

[ref264] Staudt M. (2010). Brain plasticity following early life brain injury: insights from neuroimaging. Semin. Perinatol. 34, 87–92. doi: 10.1053/j.semperi.2009.10.009, PMID: 20109976

[ref265] Staudt M. Gerloff C. Grodd W. Holthausen H. Niemann G. Krägeloh-Mann I. (2004). Reorganization in congenital hemiparesis acquired at different gestational ages. Ann. Neurol. 56, 854–863. doi: 10.1002/ana.20297 15562409

[ref266] Staudt M. Grodd W. Niemann G. Wildgruber D. Erb M. Krägeloh-Mann I. (2001). Early left periventricular brain lesions induce right hemispheric organization of speech 57, 122–125. doi: 10.1212/wnl.57.1.122, 11445639

[ref267] Staudt M. Lidzba K. Grodd W. Wildgruber D. Erb M. Krägeloh-Mann I. (2002). Right-hemispheric organization of language following early left-sided brain lesions: functional MRI topography. Neuroimage 16, 954–967. doi: 10.1006/nimg.2002.1108, PMID: 12202083

[ref268] Staudt M. Ticini L. F. Grodd W. Krägeloh-Mann I. Karnath H. O. (2008). Functional topography of early periventricular brain lesions in relation to cytoarchitectonic probabilistic maps. Brain Lang. 106, 177–183. doi: 10.1016/j.bandl.2008.01.007, PMID: 18275996

[ref269] Stavinoha P. L. Askins M. A. Powell S. K. Smiley N. P. Robert R. S. (2018). Neurocognitive and psychosocial outcomes in pediatric brain tumor survivors. Bioengineering 5:73. doi: 10.3390/bioengineering5030073 30208602 PMC6164803

[ref270] Stephens R. L. Langworthy B. W. Short S. J. Girault J. B. Styner M. A. Gilmore J. H. (2020). White matter development from birth to 6 years of age: a longitudinal study. Cereb. Cortex 30, 6152–6168. doi: 10.1093/cercor/bhaa170, PMID: 32591808 PMC7947172

[ref271] Stiles J. Reilly J. S. Levine S. C. Trauner D. A. Nass R. (2015). Neural plasticity and cognitive development: Insights from children with perinatal brain injury. Neural Plasticity and Cognitive Development Oxford, Oxford University Press.

[ref272] Stipdonk L. W. Weisglas-Kuperus N. Franken M. C. J. Nasserinejad K. Dudink J. Goedegebure A. (2016). Auditory brainstem maturation in normal-hearing infants born preterm: a meta-analysis. Dev. Med. Child Neurol. 58, 1009–1015. doi: 10.1111/dmcn.13151, PMID: 27168415

[ref273] Subramanian L. Calcagnotto M. E. Paredes M. F. (2020). Cortical malformations: Lessons in human brain development. Front. Cell. Neurosci. 13:576. doi: 10.3389/fncel.2019.00576 32038172 PMC6993122

[ref274] Tamnes C. K. Roalf D. R. Goddings A. L. Lebel C. (2018). Diffusion MRI of white matter microstructure development in childhood and adolescence: methods, challenges and progress. Dev. Cogn. Neurosci. 33, 161–175. doi: 10.1016/j.dcn.2017.12.002, PMID: 29229299 PMC6969268

[ref275] Tan K. Meiri A. Mowrey W. B. Abbott R. Goodrich J. T. Sandler A. L.. (2018). Diffusion tensor imaging and ventricle volume quantification in patients with chronic shunt-treated hydrocephalus: a matched case-control study. J. Neurosurg. 129, 1611–1622. doi: 10.3171/2017.6.JNS162784, PMID: 29350598

[ref276] Tau G. Z. Peterson B. S. (2010). Normal development of brain circuits. Neuropsychopharmacology 35, 147–168. doi: 10.1038/npp.2009.115 19794405 PMC3055433

[ref277] Taylor P. N. Kaiser M. Dauwels J. (2014). Structural connectivity based whole brain modelling in epilepsy. J. Neurosci. Methods 236, 51–57. doi: 10.1016/j.jneumeth.2014.08.010, PMID: 25149109

[ref278] ten Donkelaar H. J. Lammens M. Wesseling P. Hori A. Keyser A. Rotteveel J. (2004). Development and malformations of the human pyramidal tract. J. Neurol. 251, 1429–1442. doi: 10.1007/s00415-004-0653-3, PMID: 15645341

[ref279] Theodor K. (1907). Die Grosshirnrinde des menschen in ihren Massen und in ihrem Fasergehalt (The cerebral cortex of humans in their dimensions and their Fiber content. An Anatomical Brain Atlas). Available at: https://books.google.it/books/about/Die_Grosshirnrinde_des_Menschen_in_ihren.html?id=kqTjmgEACAAJ&redir_esc=y

[ref280] Thiel A. Vahdat S. (2015). Structural and resting-state brain connectivity of motor networks after stroke. Stroke 46, 296–301. doi: 10.1161/STROKEAHA.114.006307, PMID: 25477218

[ref281] Thompson P. M. Gledd J. N. Woods R. P. MacDonald D. Evans A. C. Toga A. W. (2000). Growth patterns in the developing brain detected by using continuum mechanical tensor maps. Nature 404, 190–193. doi: 10.1038/35004593, PMID: 10724172

[ref282] Thorbinson C. Kilday J. P. (2021). Childhood malignant brain tumors: balancing the bench and bedside. Cancers (Basel). 13:6099. doi: 10.3390/cancers13236099, PMID: 34885207 PMC8656510

[ref283] Toda K. Baba H. Ono T. Ono K. (2014). The utility of diffusion tensor imaging tractography for post-operative evaluation of a patient with hemispherotomy performed for intractable epilepsy. Brain Dev. 36, 641–644. doi: 10.1016/j.braindev.2013.08.001, PMID: 23981348

[ref284] Trevisi G. Roujeau T. Duffau H. (2016). Awake surgery for hemispheric low-grade gliomas: oncological, functional and methodological differences between pediatric and adult populations. Childs Nerv. Syst. 32, 1861–1874. doi: 10.1007/s00381-016-3069-3, PMID: 27659829

[ref285] Triplett R. L. Smyser C. D. (2022). Neuroimaging of structural and functional connectivity in preterm infants with intraventricular hemorrhage. Semin. Perinatol. 46:151593. doi: 10.1016/j.semperi.2022.151593, PMID: 35410714 PMC9910034

[ref286] Udaka Y. T. Packer R. J. (2018). Pediatric brain tumors. Neurol. Clin. 36, 533–556. doi: 10.1016/j.ncl.2018.04.009, PMID: 30072070

[ref287] Uh J. Merchant T. E. Li Y. Li X. Sabin N. D. Indelicato D. J.. (2015). Effects of surgery and proton therapy on cerebral White matter of Craniopharyngioma patients. Int. J. Radiat. Oncol. Biol. Phys. 93, 64–71. doi: 10.1016/j.ijrobp.2015.05.017 26279025 PMC5144582

[ref288] Vaessen M. J. Jansen J. F. A. Vlooswijk M. C. G. Hofman P. A. M. Majoie H. J. M. Aldenkamp A. P.. (2012). White matter network abnormalities are associated with cognitive decline in chronic epilepsy. Cereb. Cortex 22, 2139–2147. doi: 10.1093/cercor/bhr298, PMID: 22038907

[ref289] van den Heuvel M. P. Kersbergen K. J. de Reus M. A. Keunen K. Kahn R. S. Groenendaal F.. (2015). The neonatal connectome during preterm brain development. Cereb. Cortex 25, 3000–3013. doi: 10.1093/cercor/bhu095, PMID: 24833018 PMC4537441

[ref290] Verhaeghe A. Decramer T. Naets W. van Paesschen W. van Loon J. Theys T. (2018). Posterior quadrant disconnection: a Fiber dissection study. Oper Neurosurg. (Hagerstown) 14, 45–49. doi: 10.1093/ons/opx060, PMID: 29253283

[ref291] Verhelst H. Giraldo D. Vander Linden C. Vingerhoets G. Jeurissen B. Caeyenberghs K. (2019). Cognitive training in young patients with traumatic brain injury: a Fixel-based analysis. Neurorehabil. Neural. Repair 33, 813–824. doi: 10.1177/1545968319868720, PMID: 31416407

[ref292] Verhoeven J. S. Sage C. A. Leemans A. van Hecke W. Callaert D. Peeters R.. (2010). Construction of a stereotaxic DTI atlas with full diffusion tensor information for studying white matter maturation from childhood to adolescence using tractography-based segmentations. Hum. Brain Mapp. 31, 470–486. doi: 10.1002/hbm.20880, PMID: 19957267 PMC6870577

[ref293] Vulliemoz S. Vollmar C. Koepp M. J. Yogarajah M. O’Muircheartaigh J. Carmichael D. W.. (2011). Connectivity of the supplementary motor area in juvenile myoclonic epilepsy and frontal lobe epilepsy. Epilepsia 52, 507–514. doi: 10.1111/j.1528-1167.2010.02770.x, PMID: 21054353

[ref294] Weiner H. L. Placantonakis D. G. (2017). Resection of a pediatric thalamic juvenile Pilocytic astrocytoma with whole brain Tractography. Cureus 9:e1768. doi: 10.7759/cureus.1768, PMID: 29234572 PMC5724810

[ref295] Weinstein M. Ben-Sira L. Moran A. Berger I. Marom R. Geva R.. (2016). The motor and visual networks in preterm infants: An fMRI and DTI study. Brain Res. 1642, 603–611. doi: 10.1016/j.brainres.2016.04.052, PMID: 27117868

[ref296] Weinstein M. Marom R. Berger I. ben Bashat D. Gross-Tsur V. Ben-Sira L.. (2014). Neonatal neuropsychology: emerging relations of neonatal sensory-motor responses to white matter integrity. Neuropsychologia 62, 209–219. doi: 10.1016/j.neuropsychologia.2014.07.028 25090927

[ref297] Weiskopf N. Mohammadi S. Lutti A. Callaghan M. F. (2015). Advances in MRI-based computational neuroanatomy: from morphometry to in-vivo histology. Curr. Opin. Neurol. 28, 313–322. doi: 10.1097/WCO.0000000000000222 26132532

[ref298] Wendelken C. Ferrer E. Ghetti S. Bailey S. K. Cutting L. Bunge S. A. (2017). Frontoparietal structural connectivity in childhood predicts development of functional connectivity and reasoning ability: a large-scale longitudinal investigation. J. Neurosci. 37, 8549–8558. doi: 10.1523/JNEUROSCI.3726-16.2017, PMID: 28821657 PMC5577859

[ref299] Westmacott R. Askalan R. Macgregor D. Anderson P. Deveber G. (2010). Cognitive outcome following unilateral arterial ischaemic stroke in childhood: effects of age at stroke and lesion location. Dev. Med. Child. Neurol. 52, 386–393. doi: 10.1111/j.1469-8749.2009.03403.x, PMID: 19694778

[ref300] Widjaja E. Blaser S. Miller E. Kassner A. Shannon P. Chuang S. H.. (2007). Evaluation of subcortical white matter and deep white matter tracts in malformations of cortical development. Epilepsia 48, 1460–1469. doi: 10.1111/j.1528-1167.2007.01105.x, PMID: 17441991

[ref301] Widjaja E. Kis A. Go C. Raybaud C. Snead O. C. Smith M. L. (2013). Abnormal white matter on diffusion tensor imaging in children with new-onset seizures. Epilepsy Res. 104, 105–111. doi: 10.1016/j.eplepsyres.2012.10.007 23182414

[ref302] Widjaja E. Mahmoodabadi S. Z. Otsubo H. Snead O. C. Holowka S. Bells S.. (2009). Subcortical alterations in tissue microstructure adjacent to focal cortical dysplasia: detection at diffusion-tensor MR imaging by using magnetoencephalographic dipole cluster localization. Radiology 251, 206–215. doi: 10.1148/radiol.2511081092, PMID: 19190250

[ref303] Wier R. Aleksonis H. A. Pearson M. M. Cannistraci C. J. Anderson A. W. Kuttesch J. F.. (2019). Fronto-limbic white matter microstructure, behavior, and emotion regulation in survivors of pediatric brain tumor. J. Neuro-Oncol. 143, 483–493. doi: 10.1007/s11060-019-03180-5, PMID: 31073964

[ref304] Wilde E. A. Ayoub K. W. Bigler E. D. Chu Z. D. Hunter J. Wu T. C.. (2012). Diffusion tensor imaging in moderate-to-severe pediatric traumatic brain injury: changes within an 18 month post-injury interval. Brain Imaging Behav. 6, 404–416. doi: 10.1007/s11682-012-9150-y, PMID: 22399284

[ref305] Wilkinson M. Lim A. R. Cohen A. H. Galaburda A. M. Takahashi E. (2017). Detection and growth pattern of arcuate fasciculus from newborn to adult. Front. Neurosci. 11:389. doi: 10.3389/fnins.2017.00389 28769741 PMC5509799

[ref306] Williams P. T. J. A. Jiang Y. Q. Martin J. H. (2017). Motor system plasticity after unilateral injury in the developing brain. Dev. Med. Child. Neurol. 59, 1224–1229. doi: 10.1111/dmcn.13581, PMID: 28972274 PMC5773112

[ref307] Williams V. J. Juranek J. Stuebing K. K. Cirino P. T. Dennis M. Bowman R. M.. (2015). Postshunt lateral ventricular volume, white matter integrity, and intellectual outcomes in spina bifida and hydrocephalus. J. Neurosurg. Pediatr. 15, 410–419. doi: 10.3171/2014.10.PEDS13644, PMID: 25634821

[ref308] Wolfe K. R. Madan-Swain A. Kana R. K. (2012). Executive dysfunction in pediatric posterior fossa tumor survivors: a systematic literature review of neurocognitive deficits and interventions. Dev. Neuropsychol. 37, 153–175. doi: 10.1080/87565641.2011.632462, PMID: 22339228 PMC3730812

[ref309] Wu T. C. Wilde E. A. Bigler E. D. Li X. Merkley T. L. Yallampalli R.. (2011). Longitudinal changes in the Corpus callosum following pediatric traumatic brain injury. Dev. Neurosci. 32, 361–373. doi: 10.1159/000317058 PMC3073757 20948181

[ref310] Xu Y. Qiu S. Wang J. Liu Z. Zhang R. Li S.. (2014). Disrupted topological properties of brain white matter networks in left temporal lobe epilepsy: a diffusion tensor imaging study. Neuroscience 279, 155–167. doi: 10.1016/j.neuroscience.2014.08.040, PMID: 25194789

[ref311] Xu J. Rasmussen I. A. Lagopoulos J. Haberg A. (2007). Diffuse axonal injury in severe traumatic brain injury visualized using high-resolution diffusion tensor imaging. J. Neurotrauma. 24, 753–765. doi: 10.1089/neu.2006.0208 17518531

[ref312] Xue K. Luo C. Zhang D. Yang T. Li J. Gong D.. (2014). Diffusion tensor tractography reveals disrupted structural connectivity in childhood absence epilepsy. Epilepsy Res. 108, 125–138. doi: 10.1016/j.eplepsyres.2013.10.002, PMID: 24246142

[ref313] Yakovlev P. Lecours A. (1967) in The myelogenetic cycles of regional maturation of the brain. In: Resional development of the brain in early life. ed. Minkowski A. (Oxford: Blackwell), 3–70.

[ref314] Yallampalli R. Wilde E. A. Bigler E. D. Mccauley S. R. Hanten G. Troyanskaya M.. (2013). Acute White matter differences in the fornix following mild traumatic brain injury using diffusion tensor imaging. J. Neuroimaging 23, 224–227. doi: 10.1111/j.1552-6569.2010.00537.x, PMID: 21988147

[ref315] Yamao Y. Suzuki K. Kunieda T. Matsumoto R. Arakawa Y. Nakae T.. (1977). Clinical impact of intraoperative CCEP monitoring in evaluating the dorsal language White matter pathway. Hum. Brain Mapp. 38, 1977–1991. doi: 10.1002/hbm.23498 PMC6866855 28112455

[ref316] Yang J. Y. M. Beare R. Wu M. H. Barton S. M. Malpas C. B. Yeh C. H.. (2019). Optic radiation Tractography in pediatric brain surgery applications: a reliability and agreement assessment of the Tractography method. Front. Neurosci.:13:1254. doi: 10.3389/fnins.2019.01254 31824251 PMC6879599

[ref317] Yeatman J. D. Wandell B. A. Mezer A. A. (2014). Lifespan maturation and degeneration of human brain white matter. Nat. Commun. 5:4932. doi: 10.1038/ncomms5932 25230200 PMC4238904

[ref318] Yogarajah M. Duncan J. S. (2007). Diffusion-based magnetic resonance imaging and tractography in epilepsy. Epilepsia 49, 189–200. doi: 10.1111/j.1528-1167.2007.01378.x 17941849

[ref319] Yu S. Carlson H. L. Mineyko A. Brooks B. L. Kuczynski A. Hodge J.. (2018). Bihemispheric alterations in myelination in children following unilateral perinatal stroke. Neuroimage Clin. 20, 7–15. doi: 10.1016/j.nicl.2018.06.028, PMID: 29988959 PMC6034585

[ref320] Yuan W. Holland S. K. Schmithorst V. J. Walz N. C. Cecil K. M. Jones B.. (2007). Diffusion tensor MR imaging reveals persistent white matter alteration after traumatic brain injury experienced during early childhood. AJNR Am. J. Neuroradiol. 28, 1919–1925. doi: 10.3174/ajnr.A0698 17905895 PMC4295209

[ref321] Yuan W. Treble-Barna A. Sohlberg M. M. Harn B. Wade S. L. (2017). Changes in structural connectivity following a cognitive intervention in children with traumatic brain injury. Neurorehabil. Neural. Repair 31, 190–201. doi: 10.1177/1545968316675430, PMID: 27798379

[ref322] Yuan W. Wade S. L. Babcock L. (2015). Structural connectivity abnormality in children with acute mild traumatic brain injury using graph theoretical analysis. Hum. Brain. Mapp. 36, 779–792. doi: 10.1002/hbm.22664, PMID: 25363671 PMC5500248

[ref323] Zatorre R. J. Fields R. D. Johansen-Berg H. (2012). Plasticity in gray and white: neuroimaging changes in brain structure during learning. Nat. Neurosci. 15, 528–536. doi: 10.1038/nn.3045, PMID: 22426254 PMC3660656

[ref324] Zhang Z. Liao W. Chen H. Mantini D. Ding J. R. Xu Q.. (2011). Altered functional-structural coupling of large-scale brain networks in idiopathic generalized epilepsy. Brain 134, 2912–2928. doi: 10.1093/brain/awr223, PMID: 21975588

[ref325] Zhao T. Cao M. Niu H. Zuo X. N. Evans A. He Y.. (2015). Age-related changes in the topological organization of the white matter structural connectome across the human lifespan. Hum. Brain Mapp. 36, 3777–3792. doi: 10.1002/hbm.22877, PMID: 26173024 PMC6869038

[ref326] Zhu W. He J. Li X. Wang L. Lu Z. Li C.. (2017). Cognitive performance change of pediatric patients after conducting frontal transcortical approach to treat lateral ventricular tumor. Childs Nerv. Syst. 33, 2099–2108. doi: 10.1007/s00381-017-3604-x, PMID: 28939939

